# Novel *RRAGD* Variants in Autosomal Dominant Kidney Hypomagnesemia and Therapeutic Perspectives

**DOI:** 10.1016/j.ekir.2025.07.035

**Published:** 2025-07-29

**Authors:** Anastasia Adella, François Jouret, Leire Madariaga, Pieter A. Leermakers, Pedro Arango, Gema Ariceta, Bodo B. Beck, Anna Bjerre, Detlef Bockenhauer, Paula Coccia, Radhika Dhamija, Fernando de Frutos, Alejandro Garcia-Castano, Sara B. van Katwijk, Jesus Lucas, Thomas Möller, Dominik Müller, Filippo Pinto e Vairo, Melinda Raki, Jonathan Rips, Karl Peter Schlingmann, Hanka Venselaar, Matheus Vernet Machado Bressan Wilke, Tom Nijenhuis, Joost Hoenderop, Jeroen de Baaij

**Affiliations:** 1 Department of Medical BioSciences, Radboudumc, Nijmegen, The Netherlands; 2 Laboratory of Translational Research in Nephrology, Metabolism & Cardiovascular Biology, GIGA Institute, University of Liège, Liège, Belgium; 3 Division of Nephrology-Dialysis-Transplantation, University Hospital of Liége (ULiège CHU), Liège, Belgium; 4 Pediatric Nephrology Department, Biobizkaia Health Research Institute, University of the Basque Country, CIBERDEM/CIBERER, Cruces University Hospital, Barakaldo, Spain; 5 Pediatric Nephrology and Renal Transplant Department, Hospital Sant Joan de Déu, Barcelona, Spain; 6 Pediatric Nephrology Department, Vall d'Hebron Hospital, Autonomous University of Barcelona, Barcelona, Spain; 7 Faculty of Medicine, Center for Molecular Medicine Cologne, Institute of Human Genetics, University Hospital Cologne and University of Cologne, Cologne, Germany; 8 Medical Faculty, Center for Rare Diseases, University of Cologne and University Hospital Cologne, Cologne, Germany; 9 Division of Pediatric and Adolescent Medicine, Department of Transplantation and Specialized Medicine, Oslo University Hospital, Oslo, Norway; 10 Institute of Clinical Medicine, University of Oslo, Oslo, Norway; 11 Department of Paediatric Nephrology, UZ Leuven and Cellular and Molecular Physiology, KUL, Leuven, Belgium; 12 Department of Renal Medicine, University College London and Paediatric Nephrology, Great Ormond Street Hospital for Children NHS Foundation Trust, London, UK; 13 Division of Pediatric Nephrology, Hospital Italiano de Buenos Aires, Buenos Aires, Argentina; 14 Department of Clinical Genomics, Mayo Clinic, Rochester, Minnesota, USA; 15 Heart Failure and Inherited Cardiac Diseases Unit, Department of Cardiology, Hospital Universitari de Bellvitge, L’Hospitalet de Llobregat, Barcelona, Spain; 16 Bioheart Group, Cardiovascular, Respiratory and Systemic Diseases and cellular aging Program, Institut d’Investigació Biomèdica de Bellvitge (IDIBELL), L’Hospitalet de Llobregat, Spain; 17 Biobizkaia Health Research Institute, CIBERDEM/CIBERER, Barakaldo, Spain; 18 Pediatric Nephrology Department, General University Hospital of Castellón, Castellón, Spain; 19 Department of Paediatric Cardiology, Division of Paediatric and Adolescent Medicine, Oslo University Hospital, Oslo, Norway; 20 Department of Pediatric Gastroenterology, Nephrology and Metabolic Diseases, Charité, University Medicine, Berlin, Germany; 21 Department of Clinical Genomics, Center for Individualized Medicine, Mayo Clinic, Rochester, Minnesota, USA; 22 Department of Pathology, Oslo University Hospital, Oslo, Norway; 23 Department of Genetics, Hadassah Medical Center, Jerusalem, Israel; 24 Faculty of Medicine, Hebrew University of Jerusalem, Jerusalem, Israel; 25 Department of General Pediatrics, University Children’s Hospital Münster, Münster, Germany; 26 Department of Pathology and Immunology, Washington University School of Medicine, St. Louis, Missouri, USA; 27 Department of Nephrology, Radboudumc, Nijmegen, The Netherlands

**Keywords:** dapagliflozin, dilated cardiomyopathy, kidney tubulopathy, magnesium, mTORC1, RRAGD

## Abstract

**Introduction:**

Variants in the Ras-related GTPase D (*RRAGD*) gene have been associated with autosomal dominant kidney hypomagnesemia (ADKH) characterized by hypokalemia, nephrocalcinosis, and dilated cardiomyopathy (DCM). *RRAGD*, which encodes for the RagD protein, is involved in the activation of the mechanistic target of rapamycin complex 1 (mTORC1). Owing to the limited characterization of patients’ phenotypes, the understanding of *RRAGD-*associated ADKH (ADKH-RRAGD) remains incomplete. Consequently, available treatment strategies are primarily symptomatic and insufficient.

**Methods:**

In the present case series, 13 new patients and 3 novel *RRAGD* variants, that is, p.(Ser77Phe), p.(Thr91Ile), and p.(Ile100Arg), are described. To assess the pathogenicity of the novel variants, an *in vitro* assay of mTORC1 activity was performed. In addition, the clinical response to diuretics (furosemide and thiazide, *n* = 4) and Na^+^-glucose cotransporter 2 (SGLT2) inhibitor, dapagliflozin (*n* = 6) was evaluated in patients carrying the *RRAGD* p.(Thr97Pro) variant during routine.

**Results:**

The patients presented with kidney tubulopathies, including hypomagnesemia, hypercalciuria, and nephrocalcinosis. Five patients also exhibited DCM. *In vitro* assays demonstrated constitutive activation of noncanonical mTORC1 signaling caused by the p.(Ser77Phe) and p.(Ile100Arg) variants. Clinically, patients remained sensitive to diuretic challenges, whereas dapagliflozin treatment increased serum magnesium (Mg^2+^) levels by 0.04 mM but exacerbated hypokalemia.

**Conclusion:**

To date, 37 patients with ADKH-RRAGD have been identified. Kidney tubulopathy is the most prominent feature within the phenotypic spectrum of ADKH-RRAGD. Molecularly, constitutive activation of noncanonical mTORC1 is present in most *RRAGD* variants. From a therapeutic perspective, dapagliflozin may increase serum Mg^2+^ levels in patients with *RRAGD* variants.

## Graphical abstract

**Figure:**
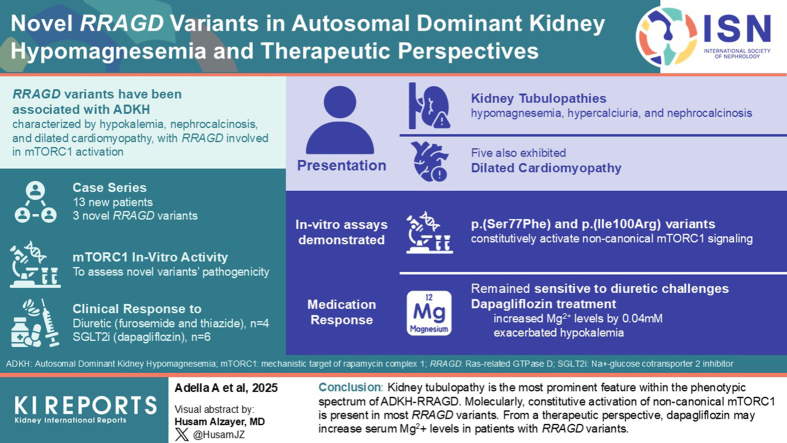

Recently, we identified gain-of-function variants in the *RRAGD* gene as the cause of ADKH, which is associated with hypokalemia, salt wasting, hypercalciuria, and nephrocalcinosis.^1^ In a subset of patients with ADKH-*RRAGD*, these kidney defects cooccurred with DCM, requiring early heart transplantation.^1^ Since our initial report, additional familial cases of ADKH-*RRAGD* have been reported.^2^^,^^3^

*RRAGD* encodes for the small GTPase RagD, one of the 4 Rag GTPases in mammalian cells (i.e., RagA-D) that serve as intracellular amino acid (AA) sensors.^4^^,^^5^ Upon AA signaling, Rag GTPases form heterodimeric complexes composed of RagA or RagB with RagC or RagD.^6^, ^7^, ^8^ In their active states, GTP-bound RagA/B and GDP-bound RagC/D recruit mTORC1 to the lysosomal surface, resulting in mTORC1 activation.^9^^,^^10^ From there, mTORC1 phosphorylates its downstream targets such as the canonical cytosolic targets, S6 kinase (S6K) and eukaryotic initiation factor 4e-binding protein 1, and its noncanonical lysosomal targets such as the transcription factor EB (TFEB).^11^, ^12^, ^13^, ^14^, ^15^, ^16^, ^17^

mTOR inhibition has been proposed as a potential treatment strategy to prevent dilated cardiomyopathy due to pathogenic *RRAGD* variants. Overexpression of the *RRAGD-associated* variants, p.(Ser76Leu) and p.(Pro119Arg) in zebrafish embryos resulted in cardiac dysfunctions.^18^ Furthermore, exposure to rapamycin, an mTOR inhibitor, rescued these phenotypes.^18^ Nevertheless, clinicians have been hesitant to prescribe lifelong mTOR inhibitors because of their immunosuppressive properties and the lack of supporting evidence for mTOR inhibitors efficiency and safety in cardiomyopathy and in patients with ADKH-RRAGD. Moreover, noncanonical mTORC1 signaling is known to be insensitive to rapamycin.^17^ Therefore, patients with stable heart function are treated with magnesium and potassium supplements, limiting their options to symptomatic treatment only. For those with heart failure and mildly reduced ejection fraction, standard therapy includes diuretics and SGLT2 inhibitors.^19^^,^^20^ Still, these patients are characterized by chronic ionic disturbances caused by a poorly understood tubular dysfunction, which might be pharmacologically improved or aggravated. This highlights the need for detailed phenotypic characterization and evaluation of diuretic response in patients with ADKH-RRAGD.

Here, we report on a new cohort of 13 patients with ADKH-RRAGD, including 3 novel *RRAGD-*associated variants, namely (p.(Ser77Phe), p.(Thr91Ile), and p.(Ile100Arg)). The functional effect of these variants was assessed by *in vitro* mTOR activity assays, TFEB translocation in T-REx HeLa cells stably overexpressing *RRAGD*-associated variants, and *in silico* RagD structure analysis. Moreover, we examined the biological response to 2 commonly used diuretics in clinical routine, that is, furosemide and hydrochlorothiazide (HCT). Finally, patients’ response to the SGLT2 inhibitor, dapagliflozin, was assessed as a potential treatment strategy in patients with ADKH-RRAGD.

## Methods

The complete methodare presented in the Supplementary Methods section.

### Study Participants

The individuals included in this manuscript were identified by routine diagnostic DNA testing (Supplementary Methods).^3^^,^^21^, ^22^, ^23^ Written informed consent was obtained for the genetic analysis and the publication of anonymized data, including the clinical challenges of diuretics.

### Molecular Assays

All *in vitro* experiments were performed using T-REx HeLa cell lines stably overexpressing *RRAGD* wild type (WT) or variants described in this study. Immunoblotting was performed on protein materials of the cells under AA stimulation. Immunocytochemistry was performed on T-REx HeLa stable cell lines transfected with pcDNA3.1-TFEB-WT-MYC (Addgene plasmid #99955).^1^ All HeLa T-REx cell lines were cultured in the culture medium described above in a humidified 37 °C incubator with 5% (v/v) CO_2_, unless stated otherwise.

### Furosemide and HCT Testing

To assess the effects of furosemide on urinary ion excretion in patients with the p.(Thr97Pro) *RRAGD* variant, a single oral dose of 40 mg furosemide was administered in routine renal physiology explorations after informed consent of each participant (*n* = 4). To assess the effects of HCT on urinary ion excretion in patients with the p.The97Pro *RRAGD* variant, a single oral dose of 50 mg HCT was administered in routine renal physiology explorations after informed consent of each participant (*n* = 4). The study duration was 6 hours postadministration of HCT. To compare the patients’ response to each diuretic to the healthy population, we reanalyzed Cl^−^ and Mg^2+^ data from furosemide testing done by Bech *et al.*^24^ using the same protocol. Mg^2+^ levels were not reported in the original publication but were taken from the unpublished study files.

### Dapagliflozin Treatment

To assess the effect of dapagliflozin on serum ion levels in patients with the p.(Thr97Pro) *RRAGD* variant (*n* = 6), blood samples were collected at baseline and 15 days after a daily oral dose of 10 mg of dapagliflozin in real-life settings.

### Statistics

For the *in vitro* studies, 2-way analysis of variance was performed. This was followed by Dunnett multiple comparisons test for the immunocytochemistry results or Šídák multiple comparisons test for the immunoblotting results. Multiple comparisons were performed by comparing the mean of mock, *RRAGD* mutants p.(Ser77Phe), p.(Thr91Ile), and p.(Ile100Arg) to the mean of *RRAGD* WT cells, within the AA treatment group. Statistical significance at *P* < 0.05 was considered significant. For other studies, no statistical tests were performed. All statistical tests were performed using GraphPad Prism version 10.4.0 for MacOS (GraphPad Software, MA). All image analyses were performed in Fiji, ImageJ2 version 2.14.0.^3^^,^^4^

## Results

### Clinical Presentation of New Patients With *RRAGD* Variants

**Figure 1:**
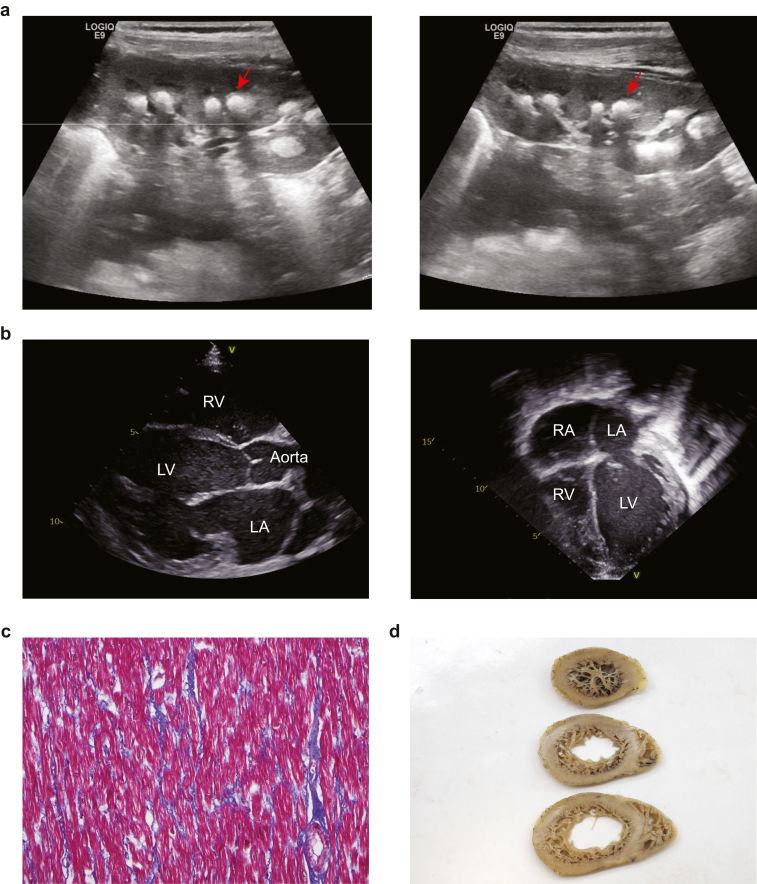
Renal tubulopathy and dilated cardiomyopathy in ADKH-RRAGD patient F7.II.1. (a and b) Ultrasound images: (a) left and right kidney showing nephrocalcinosis (structures pointed by red arrow), (b) left: parasternal long axis view of the heart, right: apical 4 chamber view of the heart. (c) Masson trichrome staining of the explanted left ventricle anterior wall longitudinal section, showing interstitial fibrosis with blue collagen fibers surrounding red individual cardiomyocytes (magnification 200x). (d) Macroscopic image of the explanted left ventricle showing prominent apical excessive trabeculation (top to bottom: apical to basal). ADKH-RRAGD, *RRAGD-*associated ADKH; LA, left atrium; LV, left ventricle; RA, right atrium; RV, right ventricle.

Routine diagnostic screening of patients with suspected familial kidney tubulopathies resulted in the identification of 8 families consisting of 13 individuals with variants in *RRAGD* (Table 1). Clinical and laboratory findings are described in Table 1, Figure 1, and Supplementary Figure S1A to C. The main kidney tubulopathy phenotypes in ADKH-RRAGD are present in all patients described in this study: hypomagnesemia, hypokalemia, salt-wasting, and nephrocalcinosis (Table 1, Figure 1a and b, Supplementary Figure S1A–C). In addition, nephrolithiasis was present in F1.III.1, F2.II.2, and F3.III.1 individuals. In family 1, the mother and 2 maternal uncles of the proband individual F1.III.1 presented with kidney tubulopathy, whereas the maternal grandmother experienced nephrolithiasis. In addition to the kidney tubulopathy, DCM was found in 5 individuals (F4.II.1, F4.II.2, F5.II.1, F7.II.1, and F8.II.2) as shown by the enlargement of left ventricles (Figure 1b, Supplementary D). Individual F4.III.3, daughter of F4.II.2, did not present with DCM but developed excessive apical trabeculations with normal left ventricular ejection fraction at the age of 6 years. Family 4 was first described by de Frutos *et al.*^3^ Heart transplantation was performed in individuals F4.II.1, F4.II.2, F5.II.1, and F7.II.1. In individual F7.1, Masson trichrome staining of explanted heart ventricular samples indicated the presence of diffuse myocardial fibrosis (Figure 1c). Moreover, extensive trabeculation was present in the apical side of the left ventricle (Figure 1d). The 7 families were described in more detail in the section Supplementary Methods.

**Table 1:** Clinical characteristics

Individuals	F1.III.1	F2.II.1	F2.II.2	F2.II.4	F3.III.1	F3.II.1	F4.II.1	F4.II.2	F4.III.3	F5.II.1	F6.II.1	F7.II.1	F8.II.2
Origin	Ashkenazi Jew	UK-SE Asia	UK-SE Asia	UK-SE Asia	Germany	Germany	Spain	Spain	Spain	Argentina	Spain	Bosnian	Ashkenazi Jew
Sex	F	F	F	M	F	F	F	F	F	M	M	F	F
Age at manifestation	Adulthood^a^	7 yr	2 yr	8 mo	15 yr	20 yr	3.5 yr	3.5 yr	6 yr	6 mo	5 yr	2.5 yr	3.1 yr
Current age	46 yr	11 yr	8 yr	1 yr	16 yr	44 yr	48 yr	48 yr	8 yr	16 yr	7.5 yr	13 yr	4.1 yr
Cardiac symptoms													
DCM (age of finding)	N	N	N	N	N	N	Y (33 yr)	Y (33 yr)	N^b^	Y	N	Y (7 yr)	Y (3.1 yr)
FS (%)	N/A	N/A	N/A	N/A	N/A	N/A	N/A	N/A	N/A	25	26	6	7
EF (%)	N/A	N/A	N/A	N/A	N/A	N/A	N/A	N/A	55	48	50	21	16
LVEDD (mm)	N/A	N/A	N/A	N/A	N/A	N/A	N/A	N/A	56	46	42	66	52
Heart transplantation (age)	N/A	N/A	N/A	N/A	N/A	N/A	Y, 47 yr	Y, 42 yr	N/A	Y, 16 yr	N/A	Y, 9 yr	N
Renal symptoms													
Hypomagnesemia-related symptoms	Y - paresthesia	N	N	N	Y - cramps, weakness		Y - carpopedal spasms	Y - carpopedal spasms	Y - paresthesia and tetany	Y	N	N	Y - weakness
Nephrocalcinosis	Y	Y	Y	Y	Y	Y	Y	Y	Y	Y	Y	Y	Y
Nephrolithiasis	Y	N	Y	N	N	Y	N	N	N	N	N	N	N
Polyuria	N	N	N	N	N	N	?	?	Y (+ polyhydramnios)	N	Y	Y	N
Metabolic alkalosis	Y	Y	Y	N	Y	n.d.	Y	Y	Y	Y	Y	Y	N
Laboratory findings													
S-Ca (mmol/l; *n* = 2.2–2.6)	2.4	2.39	2.46	2.68	2.28	n.d.	1.72	1.72	2.5	2.44	2.37	2.48	2.1
S-Cl (mmol/l; *n* = 98–107)	100	102	99	106	98		92	92	105	97	99	N/A	96
S-K (mmol/l; *n* = 3.5–5.1)	3.5	3.4	2.9	4.2	2.8		2.8	2.8	3.76	3.4	3	3	2.5
S-Mg (mmol/l; *n* = 0.7–1.1)	0.41	0.61	0.62	0.93	0.52		0.45	0.53	0.62	0.33	0.37	0.63	0.33
S-Na (mmol/l; *n* = 136–145))	139	139	137	139	141		136	135	142	136	137	134	139
S-PO_4_ (mmol/l)	1.13	1.48	1.29	2.15	0.92		1.55	1.55	1.58	1.3	1.36	1.2	4.35
S-creatinine (mg/dl; *n* = 0.73–1.18)	0.64	0.47	0.41	0.29	0.41		0.8	0.8	0.34	0.38	0.37	0.38	0.6
S-HCO_3_ (mmol/l; *n* = 22–31)	22	26	27	21	27.9		31	30	29.4	29.8	23	N/A	19.7
FE-K (%; *n* = 5.5–17)	17–77 mmol/24 h 55	13	19	11	n.d.		n.d.	n.d.	18.7	21	17	5.6	16
FE-Mg (%; *n* = 3–5)	51–269 mg/24 h 246	3.2	6.2	2.9	n.d.		n.d.	n.d.	13	14.2	16	8.50	37
FE-Na (% = 0.1–2)	41 - 227 mmol/24 h 106	0.3	0.1	0.4	n.d.		n.d.	n.d.	1.4	0.3	0.5	0.13	n.d.
Ca-to-crea ratio (mol/mol)	eGFR > 60 ≥ 60 ml/min per 1.73 m^2^	0.26	1.03	1.4			0.14 mg/mg	0.17 mg/mg	0.44 mg/mg	0.05 mg/mg	0.42 mg/mg	0.16	0.27
Therapy (specify)													
Magnesium supplementation	400 mg	N	N	N	300 mg		Y	Y	Y	Y	Y	Y	Y
Potassium supplementation	20 mEq CR	N	N	N	315 mg		Y	Y	Y	Y	Y	N	N
Heart failure medication	Eplerenone	N	N	N	N		Y	Y	N	Y	N	ACEi	Y
Others		laxatives	Laxatives, antibiotic prophylaxis (UTI)	N	Vit D 1000 I.U. per day		Immunosuppressives (heart transplant). Progression to renal failure, patient is being evaluated as a candidate for kidney transplant	immunosuppressives (heart transplant)	Citrate supplementation	Carvedilol	Potassium citrate + thiazides + enalapril	Immunosuppressive drugs: everolimus tacrolimus	Enalapril; Carvedilol; Digoxin; Spironolactone; Hydrochlorothiazide
Genetic findings (*RRAGD* variants)													
Nucleotide	c.272C>T	c.272C>T	c.272C>T	c.272C>T	c.299T>G	c.299T>G	c.227C>T	c.227C>T	c.227C>T	c.227C>T	c.227C>T	c.230C>T	c.227C>T
Protein	p.(Thr91Ile)	p.(Thr91Ile)	p.(Thr91Ile)	p.(Thr91Ile)	p.(Ile100Arg)	p.(Ile100Arg)	p.(Ser76Leu)	p.(Ser76Leu)	p.(Ser76Leu)	p.(Ser76Leu)	p.(Ser76Leu)	p.(Ser77Phe)	p.(Ser76Leu)
Inheritance	?	de novo	de novo	de novo	Dominant	Dominant	Dominant	Dominant	Dominant	de novo	de novo	de novo	de novo

Cre, creatinine; DCM, dilated cardiomyopathy; EF, ejection fraction; eGFR, estimated glomerular filtration rate; F, female; FE, fractional excretion; FS, fractional shortening; LVEDD, left ventricular end-diastolic diameter; M, male; N, no; N/A, not applicable; n.d., not determined; S-, serum value, SE, Southeast; Y, yes, ?, unknown.

a Patient F1.III.1 experienced nephrolithiasis since the age of 15 mo. Further testing was performed only in adulthood. The inheritance pattern of this patient is unknown.

b Patient F4.III.3 developed apical trabeculations at the age of 6 yrs.

**Figure 2:**
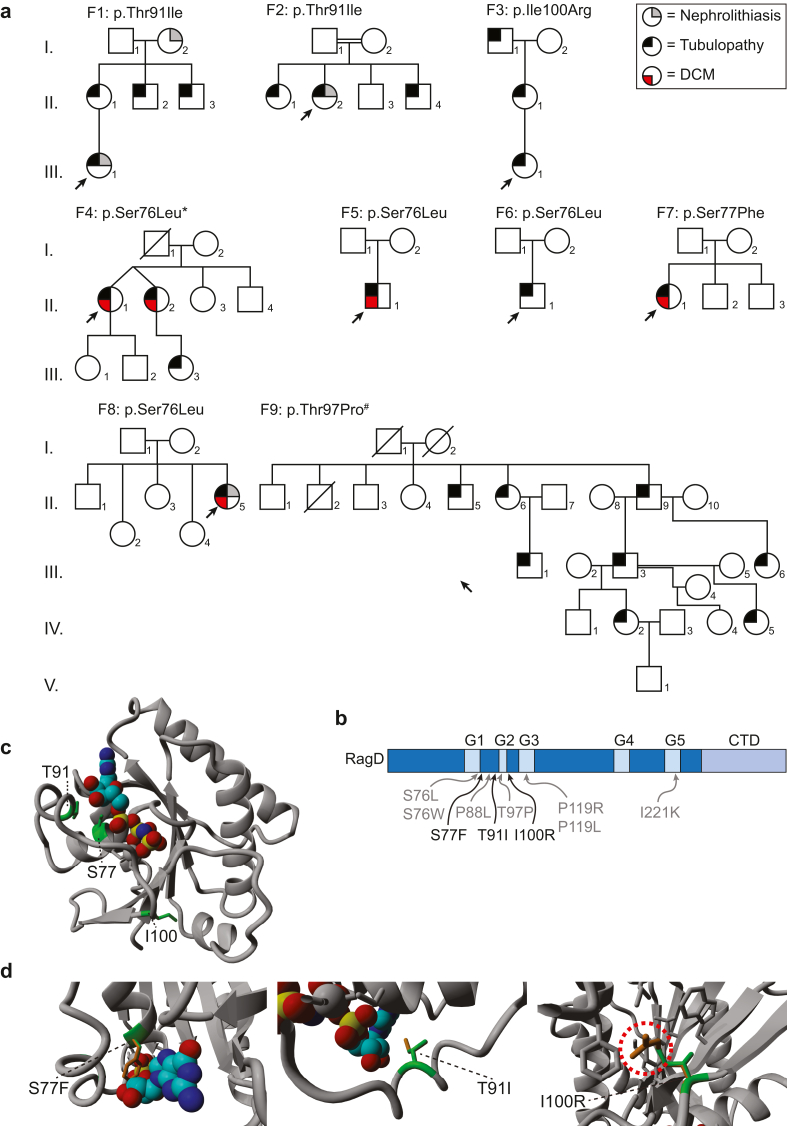
*In silico* modeling of the novel *RRAGD* variants. (a) Pedigrees of all families described in this study. The square shows males, whereas the circle shows females individuals. The crossed symbol indicates deceased individual. Filled symbols indicate affected individuals: grey indicates nephrolithiasis, black indicates kidney tubulopathy, and red indicates dilated cardiomyopathy (DCM). Proband individuals are marked with a black arrow. ∗ Adapted from de Frutos *et al.*^3 #^ Adapted from Schlingmann *et al.*^1^ and Trepiccione *et al.*^25^ (b) Schematic representation of RagD domain organization and the location of novel *RRAGD* variants (in black) and previously identified *RRAGD* variants (in grey). CTD, C-terminal domain. (c–f) Crystal structure of RagD in a complex with a GTP analog, GMPPNP (PDB structure: 2Q3F, shown as colored spheres). (c) Overview of RagD. Variant sites Ser-77 (S77), Thr-91 (T91), and Ile-100 (I100) are highlighted in green. (d) Close-up views of the mutated residues: (left) p.(Ser77Phe) (S77F), (middle) p.(Thr91Ile) (T91I), and (right) p.(Ile100Arg) (I100R). Mutated residues are highlighted in orange, the native residues are in green. The red dashed circle indicates a steric clash. F, family.

Within this patient cohort, 3 novel variants, *RRAGD* p.(Ser77Phe), p.(Thr91Ile), and p.(Ile100Arg), were identified. These variants were absent in gnomAD. For 7 individuals from 5 families (F2.II.1, F2.II.2, F2.II.4, F5.II.1, F6.II.1, F7.II.1, and F8.II.2), the *RRAGD* p.(Thr91Ie), p.(Ser76Leu) and p.(Ser77Phe) variants occurred *de novo* because both parents of the patients were unaffected (Figure 2a).

Segregation testing for p.(Thr91Ile) in the parents despite 3 out of 4 siblings affected by *RRAGD* associated hypomagnesemia in family 2 could not determine the parental origin of the *de novo* mutation. Family relationships were confirmed by microsatellite markers. In families 3 and 4, dominant inheritance was confirmed.

### *In silico* Modeling of *RRAGD* p.(Ser77Phe), p.(Thr91Ile), and p.(Ile100Arg) Variants

To evaluate the consequences of the novel *RRAGD* variants on the protein structure, we generated an *in silico* modeling. All 3 variants are located just outside the G-box domains G1 and G2 (Figure 2b), which are predicted to mediate phosphate and Mg^2+^ binding.^26^ Multiple sequence alignment analyses showed high conservation of the Ser77, Thr91, and Ile-100 residues (Supplementary Figure S1). Using the crystal structure of RagD in complex with a GTP analog (PDB: 2Q3F), the consequences of the p.(Ser77Phe), p.(Thr91Ile), and p.(Ile100Arg) variants were evaluated. Residue Ser-77 is located within the nucleotide-binding region in RagD (Figure 2c and d). Mutating Ser77 to Phe77 drastically enlarged the physical size of the residue; and therefore, would likely interfere with the nucleotide-binding capability of RagD (Figure 2d). Residue Thr91 is positioned closely to the binding site of the nucleotide (Figure 2c and e). Thus, mutation at this residue to a larger isoleucine might affect nucleotide binding (Figure 2e). Lastly, residue Ile100 is located in a hydrophobic pocket, surrounded by other hydrophobic residues (Figure 2c and f). Such hydrophobic sides are known to be energetically favorable for ligand binding.^27^ The p.(Ile100Arg) variant, however, induced a change in properties from hydrophobic isoleucine to a larger and hydrophilic arginine (Figure 2f). In addition, upon change to arginine, a steric clash to neighboring residues was observed (Figure 2f). Thus, this variant might result in protein binding instability. Of note, we compared the corresponding RagD residues to RagC in complex with Raptor, Ragulator, RagA, and TFEB (PDB: 7UX2) and found that Ile-100 residue is not directly interacting with any of these proteins.

### Noncanonical mTORC1 Signaling is Constitutively Active Because of *RRAGD* Variants

**Figure 3:**
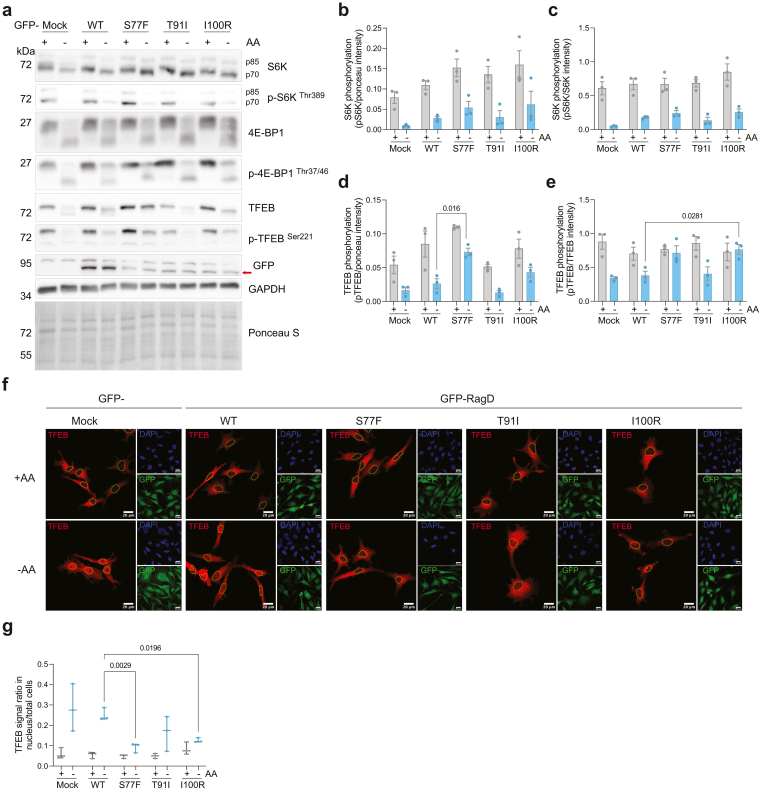
The effects of RagD p.(Ser77Phe), p.(Thr91Ile), and p.(Ile100Arg) variants on mTORC1 signaling. (a) Representative immunoblots of S6K, p-S6K, 4E-BP1, p-4E-BP1, TFEB, p-TFEB, GFP (RagD), GAPDH, and Ponceau S staining in T-REx HeLa cell lines overexpressing GFP-Mock, RagD-WT, -p.(Ser77Phe), -p.(Thr91Ile), and -p.(Ile100Arg) (S77F, T91I, and I100R in the figure, respectively) in the presence or absence of amino acids. (b–e) Graphs showing quantification of (b) p-S6K signal over ponceau, (c) p-S6K signal over S6K, (d) p-TFEB signal over ponceau, (e) p-TFEB signal over TFEB (mean ± SEM from 3 independent experiments). (f) Representative immunocytochemistry images of GFP-RagD T-REx HeLa cell lines upon TFEB transfection and subsequent AA stimulation. The cells were stained with anti-TFEB (red) and counterstained with DAPI (blue). The nuclei of TFEB-positive cells are outlined in yellow. Scale bar: 20 μm. (g) Quantification of TFEB nuclear/total cell signal from 3 independent experiments. Whiskers are extended from the maximum to the minimum points, and the middle line shows the median. 4E-BP1, 4e-binding protein 1; AA, amino acid; mTORC1, mechanistic target of rapamycin complex 1; S6K, S6 kinase; transcription factor EB, TFEB.

We have previously reported that *RRAGD* variants identified in the initial cohort resulted in the overactivation of mTORC1 signaling.^1^ To study the effects of the new variants described in this study on mTORC1 signaling, stable T-REx HeLa cell lines overexpressing GFP (mock), or GFP-RagD WT or mutants p.(Ser77Phe), p.(Thr91Ile), and p.(Ile100Arg) were generated. The cells were exposed to AA-rich or AA-deprived medium for 1 hour. Subsequently, phosphorylation of canonical and noncanonical mTORC1 targets, S6K, 4e-binding protein 1, and TFEB was assessed (Figure 3a–e). Under AA-deprived conditions, phosphorylation of TFEB in RagD-p.(Ser77Phe) and -p.(Ile100Arg) cells was significantly higher than in RagD-WT cells (mean ± SEM; p.(Ser77Phe) 0.07 ± 0.01 vs. WT 0.03 ± 0.01; p.(Ile100Arg) 0.80 ± 0.07 vs. WT 0.40 ± 0.06) (Figure 3a, d, and e). Interestingly, TFEB phosphorylation was not different in RagD-p.(Thr91Ile) cells compared with RagD-WT cells (Figure 3a–e). No significant differences in S6K and 4e-binding protein 1 phosphorylation were detected in all cell lines, both in AA-rich and AA-deprived conditions (Figure 3a–c).

Previously, TFEB was reported to be retained in the cytoplasm when RagD is constitutively active.^2^ To test if TFEB subcellular localization is affected by the 3 novel *RRAGD* variants, all GFP-RagD T-REx HeLa cell lines were transiently transfected with TFEB and exposed to AA deprivation (1 hour), after which immunocytochemistry was performed for TFEB (Figure 3f and g). In line with the immunoblotting results, overexpression of RagD-p.(Ser77Phe) and -p.(Ile100Arg) resulted in a significantly reduced nuclear translocation of TFEB under AA-deprived compared with RagD-WT cells (p.(Ser77Phe) 0.09 ± 0.01, p.(Ile100Arg) 0.13 ± 0.01 vs. WT 0.25 ± 0.02) (Figure 3f and g). In addition, RagD-p.(Thr91Ile) overexpression did not affect TFEB nuclear translocation in AA-deprived conditions (Figure 3f and g).

#### Diuretic Challenges

**Figure 4:**
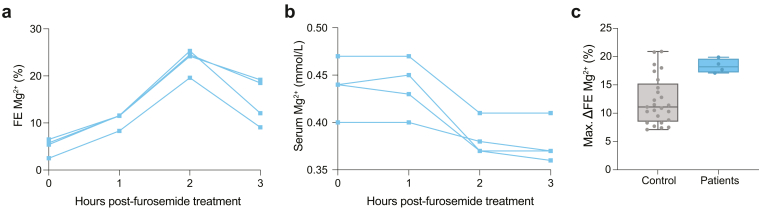
The effects of furosemide diuretics administration on fractional excretion (FE) of magnesium and serum magnesium in patients with ADKH-RRAGD. Fractional excretion of (a) Mg^2+^, (b) serum Mg^2+^ and maximal Δ FE Mg^2+^ in patients with ADKH-RRAGD (blue, *n* = 4) or healthy individuals (grey, *n* = 25)^22^ following p.o. 40 mg furosemide. (c) In the box and whiskers graph, the box represents the 25th to the 75th quartile, the whiskers extend from the minimum to maximum points, and the middle line represents the median. Data points represent individuals. ADKH-RRAGD, *RRAGD-*associated ADKH.

To further characterize the renal phenotype of the *RRAGD* variants, we examined the patients’ response to furosemide and HCT diuretics as measures for Na^+^ reabsorption in the thick ascending limb (TAL) and distal convoluted tubule, respectively. Four patients carrying the *RRAGD* p.(Thr97Pro) variant (previously reported in^1^) were tested in hospital settings for renal physiology explorations. In healthy populations, 40 mg of furosemide causes increased urinary excretion of Na^+^, K^+^, Cl^−^, Ca^2+^, and Mg^2+^.^24^^,^^28^^,^^29^ As depicted in Table 2, the median (min–max) fractional excretions (FEs, %) at baseline (T = 0) were Na^+^ (0.71 [0.1–1]), K^+^ (14.6 [6.60–26]), Cl^−^ (1.03 [0.2–2]), Ca^2+^ (0.8 [0.1–1.1]), and Mg^2+^ (5.6 [2.5–6.5]). These FEs increased over time after furosemide ingestion and peaked after 2 hours (Figure 4a, Supplementary Table S2, Supplementary Figure S2A–D). Accordingly, serum Mg^2+^ concentrations decreased from 0.44 mM (0.40–0.47) at baseline to 0.37 mM [0.36–0.41] (Figure 4b). To compare the patients’ response to furosemide to the healthy population, we reanalyzed data from Bech *et al.*.^24^ The maximal increase of FE of Cl^−^ (i.e., maximal Δ FE Cl^−^) of the patients with ADKH-RRAGD following furosemide treatment is within the range of healthy individuals (patients with ADKH-RRAGD: 13.0% [12.4–17.6] vs. healthy individuals: 11.23% [3.9–26.3]) (Supplementary Figure S3E, Table 2). The maximal Δ FE Mg^2+^ of patients with ADKH-RRAGD was in the higher portion than in healthy individuals but still falls within range (patients with ADKH-RRAGD: 18.2% [17.1–19.9] vs. healthy individuals: 11.1% [7.1–20.9]) (Figure 4c).

**Table 2:** Furosemide treatment

Furosemide treatment	Healthy controls (*n* = 25)	Patients with ADKH-*RRAGD* (*n* = 4)
Baseline FE Cl^−^ (%)	1.16 (0.45–6.74)	1.0 (0.2–2.1)
Maximal FE Cl^−^ (%)	12.79 (5.24–27.5)	14.3 (13.4–18.4)
Maximal ΔFE Cl^−^ (%)	11.23 (3.91–26.29)	13.0 (12.4–17.6)
Time max. FE Cl^−^ (h)	2 (1–3)	2 (2–2)
Baseline FE Mg^2+^ (%)	2.8 (0.3–6.3)	5.6 (2.5–6.5)
Maximal FE Mg^2+^ (%)	14.5 (7.7–25.8)	24.4 (19.6–25.3)
Maximal ΔFE Mg^2+^ (%)	11.1 (7.1–20.9)	18.2 (17.1–19.9)
Time max. FE Mg^2+^ (h)	2 (1–3)	2 (2–2)

ADKH-RRAGD, *RRAGD-*associated ADKH; FE, fractional excretion.

Fractional excretion (FE) of Cl^−^ and Mg^2+^ at baseline (T = 0), maximal, maximal Δ (maximal value − baseline value), and the time of maximal FE reached. Values represent the median [min–max] of 25 healthy individuals^22^ and 4 patients with ADKH-RRAGD following p.o. 40 mg furosemide for 3 h.

**Figure 5:**
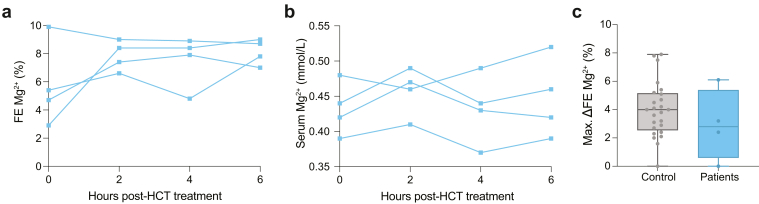
The effects of HCT diuretics administration on fractional excretion of magnesium and serum magnesium in patients with ADKH-RRAGD. Fractional excretion (FE) of (a) Mg^2+^, (b) serum Mg^2+^, and maximal Δ FE Mg^2+^ in patients with ADKH-RRAGD (blue, *n* = 4) or healthy individuals (grey, *n* = 25)^22^ following p.o. 50 mg HCT administration. (c) In the box and whiskers graph, the box represents the 25th to the 75th quartile, the whiskers extend from the minimum to maximum points, and the middle line represents the median. Data points represent individuals. ADKH-RRAGD, *RRAGD-*associated ADKH; HCT, hydrochlorothiazide.

The administration of 50 mg of HCT typically causes an increased urinary excretion of Na^+^, K^+^, and Cl^-^, with decreased Ca^2+^ excretion.^24^^,^^30^ Magnesium excretion physiologically remains unchanged or slightly increased after HCT. As summarized in Table 3, median (min–max) FEs (%) at baseline were Na^+^ (0.5 [0.2–1.3]), K^+^ (17.2 [6.3–25.5]), Cl^−^ (1.2 [0.3–2.1]), Ca^2+^ (0.6 [0.3–1]), and Mg^2+^ (5.1 [2.9–9.9]). The FEs of Na^+^, K^+^, and Cl^−^ increased in as early as 2 hours and remained stably high (Supplementary Table S3, Supplementary Figure S4A–C). FE Ca^2+^ stayed stable throughout the 6 hours (Supplementary Figure S4D). Compared with the healthy individuals cohort treated with 50 mg HCT for 6 hours,^24^ the maximal median of Δ FE Cl^−^ after HCT administration in patients with ADKH-RRAGD was similar to the healthy cohort (3.1% [2.5–4.3] patients with ADKH-RRAGD vs. 2.5% [1.3–4.7] healthy individuals) (Supplementary Figure S4E, Table 3).^22^ Furthermore, the FE of Mg^2+^ steeply increased after 2-hour oral exposure to 50 mg of HCT (24.4% [19.6–25.3] vs. 11.6% [7.7–18.8]) in healthy controls), with no significant change in serum Mg^2+^ levels (Figure 5 A and B, Table 3). The maximal median Δ FE Mg^2+^ in patients with ADKH-RRAGD, however, was in range with that in healthy individuals (2.8% [0–6.1] vs. 4% [0–7.9]) (Figure 5c, Table 3).

**Table 3:** HCT treatment

HCT treatment	Healthy controls (*n* = 25)	Patienst with ADKH-*RRAGD* (*n* = 4)
Baseline FE Cl^−^ (%)	1.04 (0.26–1.64)	1.19 (0.31–2.1)
Maximal FE Cl^−^ (%)	3.47 (2.13–5.63)	4.18 (3.65–4.65)
Maximal ΔFE Cl^−^ (%)	2.53 (1.32–4.73)	3.15 (2.46–4.29)
Time max FE Cl^−^ (h)	4 (2–4)	3 (2–4)
Baseline FE Mg^2+^ (%)	3.3 (0.1–6.3)	5.05 (2.9–9.9)
Maximal FE Mg^2+^ (%)	6.6 (4–12.9)	8.45 (7.8–9.9)
Maximal ΔFE Mg^2+^ (%)	4 (0–7.9)	2.8 (0–6.1)
Time max. FE Mg^2+^ (h)	4 (0–6)	5 (0–6.1)

ADKH-RRAGD, *RRAGD-*associated ADKH; FE, fractional excretion; HCT, hydrochlorothiazide.

FE of Cl^−^ and Mg^2+^ at baseline (T = 0), maximal, maximal Δ (maximal value – baseline value), and the time of maximal FE reached. Values represent the median [min–max] of 25 healthy individuals^22^ and 4 patients with ADKH-RRAGD following p.o. 50 mg HCT () for 6 h.

#### Therapeutic Perspective

**Figure 6:**
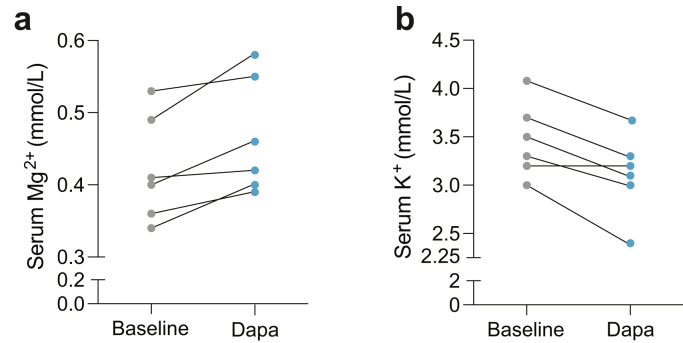
Dapagliflozin and serum Mg^2+^ and K^+^ levels in patients with ADKH-RRAGD. Serum (a) Mg^2+^ and (b) K^+^ levels at day 0 (baseline) and after 15 days of 10 mg dapagliflozin treatment (dapa) in 6 patients with ADKH-RRAGD. Data points represent individuals. ADKH-RRAGD, *RRAGD-*associated ADKH.

Current therapy available for patients with ADKH-RRAGD is limited to Mg^2+^ and K^+^ supplementation. Although SGLT2 inhibitors, including dapagliflozin, have been recommended in the management of patients with chronic cardiomyopathy,^31^ no data are available on the use of SGLT2 inhibitors in patients with ADKH-RRAGD to date. Here, we have evaluated the impact of 15 days of daily exposure to 10 mg dapagliflozin on serum ion levels in 6 patients from the p.(Thr97Pro) *RRAGD* family. After 15 days of dapagliflozin intake, serum concentration of Mg^2+^ was increased by 0.04 mM from baseline (median [min–max], 0.40 [0.34–0.53] mM) to day 15 (0.44 [0.39–0.58] mM) (Table 4, Figure 6a). Of note, the median serum K^+^ levels dropped by 0.25 mM at day 15 (3.4 [3.0–4.1] at baseline; 3.15 [2.4–3.7] at day 15) (Figure 6b). Serum creatinine levels did not change within the 15 days of dapagliflozin treatment (0.68 mg/dl [0.5–1] at baseline; 0.69 mg/dl [0.5–1] at day 15).

**Table 4:** Dapagliflozin data

Dapagliflozin data	Baseline	Dapa	Δ Dapa-baseline
Blood			
Osmolarity (mosm/kg; *n* = 281–303)	281 (281*–*291)	282 (279*–*288)	1.00
Na^+^ (mmol/l; *n* = 136*–*145)	142 (138*–*143)	141 (140*–*144)	−1.00
K^+^ (mmol/l; *n* = 3.5*–*5.1)	3.4 (3*–*4.08)	3.15 (2.4*–*3.67)	−0.25
Cl^−^ (mmol/l; *n* = 98*–*107)	96 (90*–*108)	95.5 (88*–*106)	−0.50
Ca^2+^ (mmol/l; *n* = 2.2*–*2.6)	2.4 (2.3*–*2.7)	2.44 (2.3*–*2.51)	0.04
Mg^2+^ (mmol/l; *n* = 0.66*–*1.07)	0.40 (0.34*–*0.53)	0.44 (0.39*–*0.58)	0.04
HCO3 (mmol/l; *n* = 22*–*31)	33.75 (26*–*38.4)	31.4 (27.4*–*39.6)	−2.35
Creatinine (mg/dl; *n* = 0.73*–*1.18)	0.68 (0.47*–*0.9)	0.69 (0.49*–*1)	0.01
eGFR (mL/min per 1.73 m^2^; *n* ≥ 60)	109.9 (88.1*–*128.5)	104.65 (77.6*–*128.5)	−5.25
PTH 3rd generation (ng/l)	27.9 (18.1*–*40.1)	22.9 (15.5*–*33.4)	−5.00
Glucose (mg/dl; *n* = 60*–*100)	91.5 (75*–*142)	94 (78*–*151)	2.50
Urine			0.00
Osmolarity (mosm/kg; *n* = 50*–*1200)	152.65 (88*–*214.2)	112.75 (49*–*214)	−39.90
Na^+^ (mmol/l; *n* = 22.3*–*200.1)	70.54 (42*–*82.94)	64.48 (47.3*–*95)	−6.07
K^+^ (mmol/l; *n* = 20.6*–*101.9)	100.5 (76*–*125)	174 (131*–*217)	73.50
Cl^−^ (mmol/l; *n* = 27*–*225)	3.96 (2.63*–*6.3)	4.3 (2.6*–*8.8)	0.34
Creat (mg/dl; *n* = 0*–*37.7)	5.06 (1.82*–*6.21)	3.9 (3.1*–*6.33)	−1.16
Ca/Creat (mmol/g creat; *n* = 0.3*–*6.1)	0.4 (0.4*–*0.4)	6.63 (1.6*–*27.09)	6.23
Mg/Creat (mmol/g creat; *n* = 0.74*–*4.53)	1.43 (0.7136*–*2.0)	1.31 (0.673*–*1.98)	−0.11

Creat, creatinine; eGFR, estimated glomerular filtration rate; PTH, parathyroid hormone.

Values represent the median [min; max] of 6 patients following p.o. 10 mg dapagliflozin for 15 days.

## Discussion

In this study, we identified 8 novel families comprising 13 patients with pathogenic variants in *RRAGD*. Pathogenicity of the novel *RRAGD* variants was confirmed by assessing mTORC1 activity in T-REx HeLa cells. The identified *RRAGD* p.(Ser77Phe) and p.(Ile100Arg) variants rendered mTORC1 insensitive to AA starvation, resulting in a constitutive activation of the noncanonical mTORC1 signaling pathway. In addition, diuretic challenges revealed that Na^+^ reabsorption in patients remained sensitive to furosemide and thiazide treatment. Most importantly, SGLT2 inhibition by dapagliflozin increased serum Mg^2+^ levels in a small cohort of 6 individuals with the *RRAGD* p.(Thr97Pro) variant.

RagD, one of the 4 small Rag GTPases, is involved in AA sensing by mTORC1. Using an *in vitro* model, we demonstrated that p.(Ser77Phe) and p.(Ile100Arg) *RRAGD* variants rendered mTORC1 noncanonical signaling (i.e., TFEB phosphorylation) insensitive to AA signaling, but not the canonical signaling (i.e., S6K and 4e-binding protein 1 phosphorylation). Specifically, these *RRAGD* variants led to constant phosphorylation of TFEB and reduced TFEB translocation to the nuclei. This is in line with Sambri *et al.*’s study, where noncanonical mTORC1 signaling was affected by the *RRAGD* variants in their *in vitro* model.^2^ Concerning mTORC1 canonical signaling, we previously showed that most of the *RRAGD* variants from the initial patient cohort resulted in the overactivation of canonical mTORC1 signaling as shown by increased phosphorylation of S6K under AA starvation.^1^ Indeed, our results showed that p.(Ser77Phe) and p.(Ile100Arg) *RRAGD* variants tended toward a slight increase in S6K phosphorylation, but this was not statistically significant. Therefore, we suggest that *RRAGD* variants affect noncanonical mTORC1 signaling more strongly than canonical signaling and that the degree of overactivation of the mTORC1 canonical signaling might vary per mutation, as already previously highlighted by the *RRAGD* p.(Thr97Pro) variant.^1^

Common symptoms reported in this cohort included hypomagnesemia (12/13), nephrocalcinosis (12/13), hypokalemia (11/13), metabolic alkalosis (10/13), and DCM (5/13). These 13 new patients with ADKH-RRAGD, together with previous reports, represent the most extensive phenotypic characterization of the disease to date (Table 5).^1^, ^2^, ^3^^,^^25^ Across 37 identified patients, kidney tubulopathies were nearly universal, with 24 presenting in childhood and 11 developing them later in life. In all known *RRAGD* cases, cardiomyopathies are present in about half of the patients (*n* = 18), with DCM being the most prominent form (*n* = 13). DCM was diagnosed in childhood in 9 out of the 13 affected patients. In addition to DCM, other forms of cardiomyopathy are found among patients with ADKH-RRAGD, which are ventricular arrhythmia (3/18), myocardial infarction (1/18), and excessive apical trabeculations with normal left ventricular ejection fraction (1/18). Among these cases of non-DCM cardiomyopathy, 3 patients were diagnosed in childhood and the other 2 in adulthood.

**Table 5:** Summary of all identified patients with pathogenic *RRAGD* variants

Variants	p.(Ser76Leu)	p.(Ser76Trp)	p.(Ser77Phe)	p.(Pro88Leu)	p.(Thr91Ile)	p.(Thr97Pro)	p.(Ile100Arg)	p.(Pro119Leu)	p.(Pro119Arg)	p.(Ile221Lys)	Total
Patients (no. of families)	9 (7)	1 (1)	1 (1)	8 (1)	4 (2)	8 (1)	2 (1)	1 (1)	2 (2)	1 (1)	37 (18)
Initial clinical presentation	KT (5), DCM (4)	KT (1)	KT (1)	KT (5), DCM (1)	KT (4)	KT (8)	KT (2)	DCM (1)	KT (2)	DCM (1)	KT (28), DCM (7)
Childhood tubulopathies^a^	Yes	Yes	Yes	3/8	3/4	2/8	1/2	Yes	Yes	Yes	24
Adulthood tubulopathies	No	No	No	4/8	1/4	6/8^b^	1/2	No	No	No	11
Hypomagnesemia	Yes	Yes	Yes	Yes	Yes	Yes	1/2	Yes	Yes	Yes	36
Nephrocalcinosis	Yes	Yes	Yes	3/8	Yes	No	Yes	Yes	Yes	Yes	22
Nephrolithiasis	No	No	No	3/8	2/4	1/8	1/2	?	?	No	7
Polyuria	2/9	Yes	Yes	2/8	No	2/8	No	Yes	Yes	Yes	12
Metabolic alkalosis	6/9	Yes	Yes	?	3/4	4/8	1/2	No	No	Yes	17
Childhood DCM^a^	4/9	No	Yes	No	No	No	No	Yes	Yes	Yes	9
Adulthood DCM	2/9	No	No	2/8	No	No	No	No	No	No	4
Heart transplantation	4/9 (27 yr)^c^	No	Yes (9 yr)	1/8 (43 yr)	No	No	No	No	1/2 (25 yr)	Yes (15 yr)	7 (18 yr)^c^
Other cardiomyopathies	1/9	No	No	4/8	No	No	No	No	No	No	5

DCM, dilated cardiomyopathy; KT, kidney tubulopathy; ?, unknown number of cases or has never been investigated.

Summary of known patients with pathogenic *RRAGD* variants described in this study and previous reports.^1^, ^2^, ^3^^,^^29^

a Infancy – early adolescence (£ 18 yrs).

b some family members were incidentally diagnosed in adulthood due to family screening.

c average age.

Interestingly, almost half of all patients (17/37) presented with isolated kidney tubulopathies (age range: 8–48 years), suggesting that renal and cardiac phenotypes can occur independently. Although cardiomyopathies typically manifest early, 4 patients developed DCM in adulthood. Thus, we recommend that health care providers continue to monitor the cardiac health of patients with ADKH-RRAGD, with particular attention to pediatric cases. Regarding the distribution by sex of the patients with DCM, 43% of the affected females and 21% of the affected males presented with DCM. In the future, it would be important to monitor if age and sex are determinant factors for disease outcomes and if more factors can be identified. This would require the identification of more patients and follow-up of current patients. Finally, we would like to highlight that almost half of all patients (17/37) presented with only kidney tubulopathy and no cardiac dysfunction, at least until the most recent follow-up, suggesting that the 2 key phenotypes can occur separately. Therefore, future ADKH-RRAGD screening should be done in patient cohorts with idiopathic DCM or other cardiomyopathies with proven or frequent genetic origin.

Due to the variable expressivity (differences or discrepancies in the clinical phenotype between affected individuals) and the allelic heterogeneity observed (i.e., not all variants lead to the same clinical manifestations), we hypothesized that the severity of symptoms can be attributed to the degree of mTORC1 dysregulation caused by the *RRAGD* variant.^1^ However, our data revealed that the effects of the variant on mTORC1 activation alone are not linear to the clinical manifestations. In this study, we described patients with *RRAGD* p.(Thr91Ile) exhibiting the complete set of renal phenotypes seen in other patients without DCM. *In vitro* assessment showed that this variant did not induce mTORC1 overactivation in our stable T-REx HeLa cell line. Previously, we have observed that the *RRAGD* p.(Thr97Pro) variant induced a less pronounced mTORC1 signaling activation.^1^ Interestingly, patients with p.(Ile100Arg) variant presented with the same clinical manifestations as p.(Thr91Ile) patients. However, the *RRAGD* p.(Ile100Arg) variant did show an increased mTORC1 activity under AA starvation. Nevertheless, the effects of *RRAGD* variants on mTORC1 signaling have so far been assessed in HEK293 and HeLa cells, as well as whole zebrafish embryo lysates.^1^^,^^2^^,^^18^ Future functional studies to assess the effects of *RRAGD* variants on renal transport and cardiac function are necessary to fully elucidate the molecular mechanisms of this rare disease.

The renal phenotypes seen in patients with ADKH-RRAGD resemble Bartter syndrome and familial hypomagnesemia, hypercalciuria, and nephrocalcinosis, in which the TAL section of the renal tubule is affected.^32^, ^33^, ^34^, ^35^ In family 9, which is affected by the variant, p.(Thr97Pro), the absence of hypercalciuria and nephrocalcinosis more closely resembles Gitelman syndrome, where the Na^+^-Cl^−^ cotransporter is impaired, suggesting a defect in the distal convoluted tubule.^36^ This, together with the fact that RagD is mainly expressed in the distal segments of mouse nephrons, points to a TAL and distal convoluted tubule defect in patients with ADKH-RRAGD.^1^ Paracellular Mg^2+^ and Ca^2+^ transport in the TAL is driven by the activity of the Na^+^-K^+^-2Cl cotransporter 2. Thus, dysfunctional Na^+^-K^+^-2Cl cotransporter 2 could affect Mg^2+^ and Ca^2+^ homeostasis. Our diuretics studies demonstrated that the Na^+^ and Cl^−^ reabsorption in these 2 segments is unaffected by the *RRAGD* p.(Thr97Pro) variant because the patients remained sensitive to diuretic challenges. Moreover, the change in FE of Cl^−^ induced by diuretics administration in patients with ADKH-RRAGD was comparable to the effects seen in healthy individuals administered with the same dose of diuretics and screened for the same amount of time as our patients.^24^ Of note, our study was performed during clinical routine, whereas Bech *et al.*^24^ controlled the chloride intake. Nevertheless, because patients’ response to diuretics is preserved, this suggests that Mg^2+^ and Ca^2+^ imbalances in patients with ADKH-RRAGD are not due to dysfunctions in Na^+^-K^+^-2Cl cotransporter 2 and Na^+^-Cl^−^ cotransporter but might directly target Mg^2+^ and Ca^2+^ transport in the TAL and distal convoluted tubule.

Currently, the therapeutic management of patients with ADKH-RRAGD focuses on symptomatic treatment with Mg^2+^ and K^+^ supplementation. According to guidelines, patients with DCM should also receive renin-angiotensin system and SGLT2 inhibitors.^19^^,^^20^ In this study, we explored the impact of dapagliflozin (an SGLT2 inhibitor) treatment on serum Mg^2+^ levels. We demonstrated that dapagliflozin increased serum Mg^2+^ levels in patients with *RRAGD* p.(Thr97Pro) by 10% (i.e., by 0.04 mM). Interestingly, SGLT2 inhibitors have been shown to have both renal and cardioprotective properties in both patients with and without type 2 diabetes mellitus.^37^^,^^38^ More recently, SGLT2 inhibitors have been associated with a mild increase in serum Mg^2+^ levels (0.06–0.3 mM) in patients with diabetes with or without hypomagnesemia at baseline.^39^, ^40^, ^41^ The mild increase in serum magnesium level is, however, significant because of the inherent difficulty to raise serum magnesium by oral supplementation in patients with a renal magnesium leak. In addition to increasing serum Mg^2+^ levels, in separate studies, the use of SGLT2 inhibitors reduced mTORC1 activation in the kidney and cardiac myocytes, further strengthening the potential benefit of this drug for patients with ADKH-RRAGD.^42^, ^43^, ^44^ Although it was not investigated if mTORC1 activation is dampened in patients upon dapagliflozin administration, this opens up a novel treatment option for patients with ADKH-RRAGD with hypomagnesemia, DCM, and mTORC1 overactivation. However, the associated serum K^+^ reduction (median: 0.25 mM) warrants consideration when prescribing this treatment.

There are a few limitations in this study. First, HeLa cells lack the expression of relevant renal transporters, such as the claudins and Na^+^-K^+^-2Cl cotransporter 2, making functional studies not feasible in these cells. Here, we have provided evidence that mTORC1 activation is not linear to the clinical manifestations in HeLa cells in patients. It is, therefore, crucial to investigate how *RRAGD* variants affect renal ion transports and cardiac functions to further unravel the molecular mechanisms underlying this disease. Second, the dapagliflozin trial was conducted in only 1 family. As a part of the clinical workup, it should be emphasized that we did not control for the patients’ dietary intake. Future studies should evaluate the efficacy of SGLT2 inhibitors in larger cohorts with diverse *RRAGD* variants. Importantly, dapagliflozin has already been clinically tested for managing refractory hypomagnesemia and chronic cardiomyopathy.^31^^,^^40^

In conclusion, we report on a large cohort of patients with ADKH-RRAGD comprising 13 individuals with 3 novel *RRAGD* variants and present the most comprehensive phenotypic characterization of this disease to date. This work highlights the potential of SGLT2 inhibitors as a novel treatment option for patients with ADKH-RRAGD, particularly those with hypomagnesemia, DCM, and mTORC1 overactivation. Future studies should focus on elucidating the mechanisms of SGLT2 inhibitors and further assessing their therapeutic benefits in patients with ADKH-RRAGD.

## Disclosure

All the authors declared no competing interests.

## Supplementary Material

Supplementary File (PDF) Detailed clinical descriptions. **Supplementary Methods. Supplementary References. Figure S1.** Multiple *RRAGD* sequences alignment. **Figure S2.** Ultrasound images. **Figure S3.** Fractional excretions after furosemide. **Figure S4.** Fractional excretions after HCT. **Figure S5.** Uncropped immunoblots. **Table S1.** List of primers. **Table S2.** Laboratory values following furosemide treatment. **Table S3.** Laboratory values following HCT treatment.
