# Supplementary material for: Novel RRAGD Variants in Autosomal Dominant Kidney Hypomagnesemia and Therapeutic Perspectives
Source: Kidney Int Rep. 2025 Jul 29;10(10):3640–55. doi: 10.1016/j.ekir.2025.07.035 (PMC12545939; doi:10.1016/j.ekir.2025.07.035)
Supplement: Supplementary File (PDF) — Detailed clinical descriptions. Supplementary Methods. Supplementary References. Figure S1. Multiple RRAGD sequences alignment. Figure S2. Ultrasound images. Figure S3. Fractional excretions after furosemide. Figure S4. Fractional excretions after HCT. Figure S5. Uncropped immunoblots. Table S1. List of primers. Table S2. Laboratory values following furosemide treatment. Table S3. Laboratory values following HCT treatment. [file mmc1.pdf]

Supplementary appendix

Novel *RRAGD* variants in autosomal dominant kidney hypomagnesemia and therapeutic perspectives

Anastasia Adella[1], François Jouret[2,3], Leire Madariaga[4], Pieter A. Leermakers[1], Pedro Arango[5], Gema Ariceta[6], Bodo B. Beck[7,8], Anna Bjerre[9,10], Detlef Bockenhauer[11,12], Paula Coccia[13], Radhika Dhamija[14], Fernando de Frutos[15,16], Alejandro Garcia-Castano[17], Sara B. van Katwijk[1], Jesus Lucas[18], Thomas Möller[19], Dominik Müller[20], Filippo Pinto e Vairo[21], Melinda Raki[22], Jonathan Rips[23,24], Karl Peter Schlingmann[25], Hanka Venselaar[1], Matheus Vernet Machado Bressan Wilke[26], Tom Nijenhuis[27], Joost Hoenderop[1], Jeroen de Baaij[1,*]

* Corresponding author:

Jeroen de Baaij

Department of Medical BioSciences,

Radboudumc

P.O. Box 9101, 6500HB, Nijmegen, The Netherlands

Phone: +31- 24 361 7347, Email: jeroen.debaaij@radboudumc.nl

[1] Department of Medical BioSciences, Radboudumc, Nijmegen, The Netherlands

[2] Laboratory of Translational Research in Nephrology, Metabolism & Cardiovascular Biology, GIGA Institute, University of Liège, Liège, Belgium

[3] Division of Nephrology-Dialysis-Transplantation, University Hospital of Liége (ULiège CHU), Liège, Belgium

[4] Pediatric Nephrology Department, Biobizkaia Health Research Institute, University of the Basque Country, CIBERDEM/CIBERER, Cruces University Hospital, Barakaldo, Spain

[5] Pediatric Nephrology and Renal Transplant Department, Hospital Sant Joan de Déu, Barcelona, Spain

[6] Pediatric Nephrology Department, Vall d'Hebron Hospital, Autonomous University of Barcelona, Barcelona, Spain

[7] Institute of Human Genetics, Center for Molecular Medicine Cologne, University Hospital Cologne and University of Cologne, Faculty of Medicine, Cologne, Germany.

[8] Center for Rare Diseases, Medical Faculty, University of Cologne and University Hospital Cologne, Cologne, Germany.

[9] Department of Transplantation and Specialized Medicine, Division of Pediatric and Adolescent Medicine, Oslo University Hospital, Oslo, Norway

[10] Institute of Clinical Medicine, University of Oslo, Oslo, Norway

[11] Department of Paediatric Nephrology, UZ Leuven and Cellular and Molecular Physiology, KUL, Leuven, Belgium

[12] Department of Renal Medicine, University College London and Paediatric Nephrology, Great Ormond Street Hospital for Children NHS Foundation Trust, London, UK

[13] Division of Pediatric Nephrology, Hospital Italiano de Buenos Aires, Buenos Aires, Argentina

[14] Department of Clinical Genomics, Mayo Clinic, Rochester, Minnesota, USA

[15] Heart Failure and Inherited Cardiac Diseases Unit, Department of Cardiology, Hospital Universitari de Bellvitge, L'Hospitalet de Llobregat, Barcelona, Spain

[16] Bioheart Group, Cardiovascular, Respiratory and Systemic Diseases and cellular aging Program, Institut d'Investigació Biomèdica de Bellvitge (IDIBELL), L'Hospitalet de Llobregat, Spain

[17] Biobizkaia Health Research Institute, CIBERDEM/CIBERER, Barakaldo, Spain

[18] Pediatric Nephrology Department, General University Hospital of Castellón, Castellón, Spain

[19] Department of Paediatric Cardiology, Division of Paediatric and Adolescent Medicine, Oslo University Hospital, Oslo, Norway

[20] Department of Pediatric Gastroenterology, Nephrology and Metabolic Diseases, Charité, University Medicine, Berlin, Germany

[21] Center for Individualized Medicine and Department of Clinical Genomics, Mayo Clinic, Rochester, Minnesota, USA

[22] Department of Pathology, Oslo University Hospital, Oslo, Norway

[23] Department of Genetics, Hadassah Medical Center, Jerusalem, Israel

[24] Faculty of Medicine, Hebrew University of Jerusalem, Israel

[25] Department of General Pediatrics, University Children`s Hospital Münster, Münster, Germany

[26] Department of Pathology and Immunology, Washington University School of Medicine, St. Louis, Missouri, USA

[27] Department of Nephrology, Radboudumc, Nijmegen, The Netherlands

Content:

1. Supplementary Methods
2. Detailed clinical descriptions
3. Supplementary table S1: list of primers
4. Supplementary table S2: laboratory values following furosemide treatment
5. Supplementary table S3: laboratory values following HCT treatment
6. Supplementary figure S1: multiple *RRAGD* sequences alignment
7. Supplementary figure S2: ultrasound images
8. Supplementary figure S3: fractional excretions after furosemide
9. Supplementary figure S4: fractional excretions after HCT
10. Supplementary figure S5: uncropped immunoblots
11. Supplementary references

Supplementary Methods

Sequencing

Family 1

Exome sequencing was conducted at a CLIA-certified and CAP-accredited laboratory using Illumina (Illumina, California, USA) short reads platform. Manual analysis of the raw sequencing files, was done utilizing commercial genomic prioritization tools that operate through AI-driven graphical interfaces, requiring the input of VCF or BAM files, along with information on sex, age of onset, and Human Phenotype Ontology (HPO) terms. [1]

Family 2

Genomic DNA from peripheral blood leukocytes of siblings F2II.1 and F2II.2 (the offspring of a consanguineous union) previously sequenced with unremarkable yield for suspected Bartter syndrome/autosomal recessive kidney disease were resequenced. Whole exome sequencing was performed using the Agilent SureSelectXT HS Human all exon V8 enrichment (Agilent Technologies, Santa Clara, CA, USA), followed by NGS on an Illumina NovaSeq 6000 platform (Illumina, San Diego, CA, USA). Variant analysis was priorized for a custom set of 500 known rare kidney diseases (RKD V11) and yielded the heterozygous *RRAGD* variant, the only shared variant in a dominantly inherited RKD gene. Deep sequencing of genomic DNA and cellfree (cf)DNA of parental blood samples was performed by an amplicon-based approach. Coverage was 10758x (maternal) respectively 9797x (paternal) for genomic DNA and 13305x (maternal) respectively 20643x (paternal) for cfDNA.

Family 4

Genomic DNA was extracted from peripheral blood leukocytes using the MagPurix instrument (Zinexts Life Science Corp., New Taipei City, Taiwan, R.O.C.). DNA purity and concentration were determined using Qubit 2.0 fluorometer (Thermo Fisher Scientific). Library preparation was done using the Ion AmpliSeq™ Exome RDY Library Preparation kit (Thermo Fisher Scientific) according to manufacturer's instructions. Samples were then sequenced using the Ion GeneStudio S5 System (Thermo Fisher Scientific, California, USA). Base calling, read filtering, alignment to the reference human genome GRCh37/hg19, and variant calling were done using Ion Torrent Suite and Ion Reporter Software (Thermo Fisher Scientific, California, USA). Not appropriately covered amplicons (<20x) and candidate variants were assessed by Sanger sequencing after polymerase chain reaction (PCR), sequenced with fluorescent dideoxynucleotides (BigDye Terminator v3.1 Cycle Sequencing Kit, Life Technologies, Grand Island, NY, USA), and loaded onto an ABI3130xl Genetic Analyzer (Thermo Fisher Scientific, California, USA).

Families 5 and 6

Genomic DNA was extracted from peripheral blood leukocytes using the MagPurix instrument (Zinexts Life Science Corp., New Taipei City, Taiwan, R.O.C.). DNA purity and concentration were determined using Qubit 2.0 fluorometer (Thermo Fisher Scientific, California, USA). Library preparation was done using the Illumina DNA Prep with Exome 2.0 Plus Enrichment according to manufacturer's instructions. Samples were then sequenced using the NovaSeq6000 platform (Illumina, California, USA). Alignment and variant calling was carried out using Dragen 4.2.4 pipeline (Illumina, California, USA) against GRCh38 (hg38). Annotation and interpretation of identified variants was done with the Illumina Variant Interpreter Version 2.16. The variant

filtering (SNV/Indel) was performed by using pre-established filters: coding and flanking intronic variants (+/-10 bp), more than 20X read depth and less than 0.01% population frequency (GnomAD v2.1.1, https://gnomad.broadinstitute.org/). Novel DNA variant was named according to the Human Genome Variation Society guidelines (www.hgvs.org), and classified according to ACMG-AMP (American College of Medical Genetics and Genomics and the Association for Molecular Pathology) guidelines. Candidate variants were assessed by Sanger sequencing after polymerase chain reaction (PCR), sequenced with fluorescent dideoxynucleotides (BigDye Terminator v3.1 Cycle Sequencing Kit, Life Technologies, Grand Island, NY, USA), and loaded onto an ABI3130xl Genetic Analyzer (Thermo Fisher Scientific, California, USA).

Family 7

Next Generation Sequencing of relevant genes in Illumina's Trusight Cardio kit (Illumina, California, USA) was performed. Results of the sequencing revealed no known variants that cause dilated cardiomyopathy. Next, genome sequencing was performed, which included analysis of copy numbers, with filtration for variants of genes that are part of the gen panel for renal tubular disease (v2). On request from the clinician, *RRAGD* was part of the analysis.

Family 8

Trio-whole genome sequencing was conducted at the laboratory of the Genetic Institute at the Tel Aviv Medical Center. A genomic library was prepared using the Illumina PCR-Free Prep DNA protocol. Secondary analysis of the raw sequencing files (FASTQ) and tertiary analysis of the VCF files were performed using the Genoox software. The subject’s sequences were aligned to the

human genome version hg19. Variant pathogenicity classification was performed according to the ACMG 2021 guidelines and standards.

Study participants

The individuals included in this manuscript were identified by routine diagnostic DNA testing (see details below). All patients had a clinical presentation of kidney tubulopathy and genetic screening resulted in the identification of class 3-5 *RRAGD* variants. GeneMatcher was employed to find and connect multiple cases.[21] Written informed consent was obtained for the genetic analysis and the publication of anonymized data, including the clinical challenges of diuretics.

Genetic analysis

Allele frequencies of the newly identified *RRAGD* (NM_021244.5) variants were checked in the GnomAD v4.1 [23] database in July 2024.

***In silico* analysis**

For multiple sequence alignment analysis, the following sequences were used: Saccharomyces cerevisiae (UniProtKB ID: P53290), Caenorhabditis elegans (UniProtKB ID: G5EGB3), Drosophila melanogaster (UniProtKB ID: Q7K519), Danio rerio (UniProtKB ID: F1QIB5), Xenopus tropicalis (UniProtKB ID: Q0VFL0), Mus musculus (UniProtKB ID: Q7TT45), and Homo sapiens (UniProtKB ID: Q9NQL2). Multiple alignment analysis was performed using Clustal Omega (EMBL-BI).[27] To model the variants, a publicly available crystal structure of RagD in complex with a GTP analogue, Gmppnp, was used (PDB: 2Q3F). Analysis and visualization were done in YASARA.[28]

Cloning and plasmids

Human *RRAGD* construct (VectorBuilder GmbH, Neu-Isenburg, Germany (Vector ID: VB171030-1104zuv)) was amplified by PCR and subcloned into the pcDNA5/FRT/TO eGFP expression vector. The full *RRAGD* cDNA length was amplified using primers. Subsequently, the PCR product and the plasmid backbone were digested using restriction enzymes KpnI and XhoI (New England Biolabs, the Netherlands). The digested sequence was then ligated into the digested backbone behind eGFP sequence using T4 DNA ligase (New England Biolabs, the Netherlands). Subsequently, site-directed mutageneses were performed on the created pcDNA5/FRT/TO-eGFP-h*RRAGD* wild-type (WT) plasmid to obtain the h*RRAGD* p.Ser77Phe, p.(Thr91Ile), and p.(Ile100Arg) variant constructs using the QuikChange II XL site-directed mutagenesis kit (Agilent Technologies, USA). All obtained constructs were checked through Sanger sequencing for the full-length DNA. All primers used are available in Supplementary table S1. pcDNA3.1-TFEB-WT-MYC was a gift from James Brugarolas (Addgene plasmid #99955).[29]

Antibodies

The following antibodies (Cell Signaling Technology, Massachusetts, USA) were used: p70-S6K (#2708S, IB: 1:1,000), p-p70-S6K^{Thr389} (#9234S, IB: 1:1,000), TFEB (#4240S, IB: 1:1,000, ICC: 1:200) , p-TFEB^{Ser211} (#37681S**,** IB: 1:1,000), 4E-BP1 (#9452S, IB: 1:1,000), p-4E-BP1$^{Thr37/46}$ (#2855S, IB: 1:1000). Additionally, anti-RagD (#NBP2-32106, IB: 1:2,000, Novius Biologicals, Colorado, USA), anti-GFP (#G1544-100UG, IB: 1:5,000, Sigma Aldrich, Missouri, USA) and anti-GAPDH (#AM4300, IB: 1:4,000, Thermo Fisher Scientific Baltics UAB, Vilnius, Lithuania) antibodies were used. For

immunoblotting, the following peroxidase- (PO) conjugated secondary antibodies were used: anti-IgG mouse (#145-515-035, 1:10,000, Jackson ImmunoResearch Europe Ltd., Exeter, UK), anti-IgG rabbit (#A4914, 1:10,000, Merck Life Science N.V, Amsterdam, The Netherlands). For immunocytochemistry experiments, we applied a secondary anti-IgG rabbit antibody conjugated to Alexa Fluor 594 (#A11012, 1:300, Thermo Fisher Scientific, Orlando, USA).

Stable cell line generation and culture

300,000 T-REx^{TM} HeLa cells (Invitrogen, Massachusetts, USA) were seeded into a 6-well plate. 6 hr later, the cells were co-transfected with the pOG44 plasmid (Invitrogen, Massachusetts, USA), and either empty pcDNA5/FRT/TO-eGFP (mock), pcDNA5/FRT/TO-eGFP h*RRAGD* WT, or h*RRAGD* mutants, at 1:1 ratio, totaling to 2 µg DNA. As a negative control, transfection with only 2 µg of pOG44 plasmid was used. 2 µL Lipofectamine 2000 (Invitrogen, Massachusetts, USA) was used per 1 µg of DNA. After ± 18 hr, transfected cells from each well were transferred to 10-cm petri dish and left to attach for approximately 6 hr. Subsequently, 3 µg/mL blasticidine (Sigma-Aldrich, Missouri, USA) and 100 µg/mL Hygromycin B (Thermo Fisher Scientific, California, USA) was added to the cell culture medium (Dulbecco's Modified Eagle Medium (DMEM) containing 25 mM Hepes, 4.5 g/L glucose, and 4 mM L-glutamine (#42430082, Thermo Fisher Scientific, California, USA), supplemented with 10% FBS (Greiner Bio-One, Alphen aan den Rijn, the Netherlands), 1 mM sodium pyruvate (Thermo Fisher Scientific, California, USA), and 1% v/v MEM non-essential amino acids solution 100x (Capricorn Scientific GmbH, Ebsdorfergrund, Germany)) to start the selection procedure. Cell culture medium containing the antibiotics, now termed as selection medium, was replaced every 2 days until cell sorting.

9 days after the transfection, expression of GFP or fusion GFP-RagD sequence was induced in the transfected cells by adding 1 µg/mL tetracycline to the selection medium. The following day (after 24 hr), the cells were prepared for single-cell sorting using fluorescence-activated cell sorting (FACS) by the flow cytometry facility at Radboudumc. In short, on the sorting day, cells were disassociated from the Petri dishes using trypsin-EDTA solution (0.05% trypsin (w/v), 0.02% (w/v) EDTA; Sigma-Aldrich, USA) and resuspended in 1x PBS and 2% (w/v) EDTA. GFP-positive cells were sorted individually into a single 96-well plate each using 100 µm nozzle of the Cytek™ Aurora CS System (Cytek, the Netherlands). Following the cell sorting, cells were kept in the incubator to let individual clones grow. After 10 days, grown clones from each cell lines were expanded and genotyped using the subcloning primers. For each mock, RagD-WT, - p.(Ser77Phe), p.(Thr91Ile), or p.(Ile100Arg) cell line, one clone was selected and used for subsequent experiments.

Cell culture and treatments for experiments

All HeLa T-REx cell lines were cultured in the culture medium described above in a humidified 37°C incubator with 5% (v/v) CO_2 unless stated otherwise.

For immunoblotting experiments, 200,000 HeLa T-REx cells were seeded into a 6-well plate. The next day, expression of GFP or GFP-RagD was induced by adding 1 µg/mL tetracycline to the culture medium. For immunocytochemistry experiments, ± 300,000 cells were seeded into a 6-well plate. After 6 hr, cells were transfected with 0.5 µg of pcDNA3.1-WT-TFEB-MYC using FuGENE HD Transfection Reagent (Promega, Shanghai, China) at a 1:2 DNA:FuGENE ratio. The next day, these transfected cells were collected, and 20,000 cells were re-seeded on poly-L-lysine-coated

10 mm cover slips in 24-well plates. After 24 hr, tetracycline was added to the culture medium to induce the expression of GFP or GFP-RagD.

Amino acids (AA) starvation was performed 24 hr after tetracycline induction. Nutrient-rich (+AA) and starvation (-AA) media were made following the DMEM culture medium manufacturer's formulation. The -AA medium was prepared by omitting the addition of any AA's to the medium. The media were then pH-adjusted to pH 7.4 using 1 M HCl, and sterile filtered through a 0.22 µm filter device. Before use, treatment media were supplemented with 10% (v/v) dialysed FBS (Thermo Fisher Scientific, California, USA) and 1 mM sodium pyruvate. Specifically, to +AA medium, 4 mM L-glutamine (Thermo Fisher Scientific, California, USA) and MEM non-essential amino acids solution (Capricorn Scientific GmbH, Ebsdorfergrund, Germany) were added. The cells were washed with PBS, and incubated with +AA or -AA medium for 1 hr at 37°C.

Cell lysis

Cells were washed with ice-cold PBS buffer and lysed in 200 µL Triton lysis buffer (50 mM Tris-HCl pH 7.5, 1 mM EGTA, 1 mM EDTA, 1% (v/v) Triton X-100, 10 mM Na-glycerophosphate, 50 mM NaF, 10 mM Na-pyrophosphate, 270 mM sucrose, and 150 mM NaCl) supplemented with phosphatase inhibitor (1 mM Na-orthovanadate) and protease inhibitors (1 mM PMSF, 1 µM aproptin, 0.01 mM leupeptin, 1.4 µM pepstatin) by scraping on ice. Next, samples were centrifuged at 18,000xg at 4°C for 10 minutes and the supernatant was collected. Protein concentration was measured using the Pierce BCA protein assay kit (Thermo Scientific, USA) and 1 µg/µL samples were prepared in Laemmli (60 mM Tris-HCl pH 6.8, 2% (v/v) SDS, 0.01% (v/v) bromophenol blue, 6%

(v/v) glycerol, 0.1 M DTT) loading buffer. Finally, samples were denatured at 95°C for 5 mins and stored at -20°C.

Immunoblotting

Using denatured samples in Laemmli + DTT, 15 µg of protein was loaded per sample in a 12% (v/v) polyacrylamide gel and resolved through SDS-PAGE run at 50 V for 30 mins, and then 100 V for 60 mins. After this, proteins were transferred to polyvinylidene fluoride (PVDF) membranes at 100 V for 120 mins. After the transfer, similar protein loading was confirmed through Ponceau S staining (Thermo Fisher Scientific, Illinois, USA) and imaged using Gel Doc EZ Imager (Bio-Rad, California, USA). Next, the membranes were blocked in 5% (w/v) non-fat dry milk in Tris-buffered saline (TBS) containing 0.1% (v/v) Tween-20 (TBS-T; VWR, Amsterdam, the Netherlands) for 1 hr. After 1 hr, membranes were incubated in primary antibody diluted in 1% (w/v) non-fat dry milk in TBS-T at 4°C. The next day, membranes were washed 3x in TBS-T for 10 mins each, and incubated in secondary antibody diluted in 1% milk in TBS-T at RT for 1 hr. After this, the membranes were washed 3x in TBS-T and 1x in TBS for 10 mins each. To visualize the protein of interest, membranes were incubated with SuperSignal West Pico PLUS Chemiluminescent Substrate (Thermo Scientific, Illinois, USA) or SuperSignal West Femto Maximum Sensitivity Substrate (Thermo Scientific, Illinois, USA), and then imaged using the ImageQuant™ LAS 4000 (GE Healthcare, Freiburg, Germany).

To analyze the signal intensity, Fiji, ImageJ2 version 2.14.0 was used.[30,31] To correct for protein loading, Ponceau S signal was used as the reference.[32] Ponceau S signal was quantified in the

same way as immunoblot quantification with the addition of background correction using the rolling ball method using a similar radius for all independent experiments.

Immunocytochemistry

Cells were fixated with 4% (v/v) paraformaldehyde (Sigma-Aldrich) in PBS for 10 mins at RT and permeabilized with PBS containing 0.3% (v/v) Triton X-100 and 0.1% (w/v) bovine serum albumin, also for 10 mins. Next, to quench free aldehyde, samples were incubated in 50 mM NH_4Cl in PBS for 10 mins. Following 2x rinsing with PBS, samples were blocked in goat serum dilution buffer (GSDB) containing 16% (v/v) goat serum and 0.3% (v/v) Triton X-100 in PBS for 30 mins. Subsequently, samples were incubated overnight at 4°C in primary antibodies diluted in GSDB. The next day, samples were washed 3x with PBS for 10 mins each, and incubated with secondary antibodies dilutes in GSDB for 45 mins at room temperature. Samples were then washed 3x with PBS for 10 mins each. During the second wash, DAPI was added to the PBS to a final concentration of 300 nM. Finally, samples were mounted with Fluoromont-G (Southern Biotech, Alabama, USA). Images were taken with an SP8 confocal microscope with a tunable pulsed white light laser (WLL) and a fixed 405 nm laser (Leica Microsystems, Amsterdam, the Netherlands). A 63x water-based objective (NA 1.20) was used. DAPI was excited with the 405 nm diode laser with 5% laser intensity, excited at 432-474 nm wavelength, detected with PMT detector, 1000% gain. GFP acquisition parameters were: 488 argon laser with 25% intensity, 493-560 nm wavelength, HyD2 detector, and 50% gain. Alexa Fluor 594 acquisition parameters were: 594 laser with 3% intensity, 600-668 nm wavelength, HyD4 detector, and 10% gain.

To quantify TFEB nuclear translocation, an automated script was created in ImageJ2 version 2.14.0. In short, using the TFEB channel, TFEB-transfected cells were manually selected. The DAPI channel was used to determine the location and outline of the nuclei. Next, a ratio of the TFEB signal in the nuclei over the total TFEB signal in cells per image was calculated, thus, depicting the TFEB translocation to the nuclei. In each experiment, 10 images from 10 independent fields containing at least 2 TFEB-transfected cells were obtained per genotype. In the end, the average of the 10 images per genotype was calculated.

Furosemide and thiazide testing

In order to assess the effects of furosemide on urinary ion excretion in patients with the p.(Thr97Pro) *RRAGD* variant, a single oral dose of 40 mg furosemide was administered in routine renal physiology explorations after informed consent of each participant (n=4). The study duration was 3 hr *post*-administration of furosemide. Participants were in non-fasting conditions at baseline. After furosemide administration, participants remained fasting, with *ad libitum* access to water. The first post-furosemide blood sampling and urine collection occurred at 1-hr post-furosemide intake. Subsequent samplings took place at 2 hr and 3 hr post-furosemide intake. To compare the patients' response to furosemide to the healthy population, we re-analyzed Cl^- and Mg^{2+} data from furosemide testing done by Bech et al. using the same protocol (2017).[33] For our analysis, we included healthy individuals of all age ranges. Mg^{2+} levels were not reported in the original publication but were taken from the unpublished study files.

To assess the effects of hydrochlorothiazide (HCT) on urinary ion excretion in patients with p.The97Pro *RRAGD* variant, a single oral dose of 50 mg HCT was administered in routine renal

physiology explorations after informed consent of each participant (n=4). The study duration was 6 hr post-administration of HCT. Participants were in non-fasting conditions at baseline. After HCT administration, participants had *ad libitum* access to water. From T=1 (*i.e.,* 2 hr post-HCT intake), participants had the option to consume crackers, but no other food or beverages were permitted. Blood and urine samples were collected at 2, 4, and 6 hr post-HCT intake. To compare the patients' response to HCT to the healthy population, we re-analyzed Cl^- and Mg^{2+} data from thiazide testing done by Bech et al. using the same protocol (2017).[33] Healthy individuals from all age ranges were included in this study. Mg^{2+} levels were not reported in the original publication but were taken from the unpublished study files.

Detailed Clinical Descriptions

Family 1

A 46-year-old female of Ashkenazi Jewish descent with nephrolithiasis, hypomagnesemia, and hypokalemia. She has family history of the maternal grandmother experiencing nephrolithiasis, and her mother and two maternal uncles exhibiting electrolyte imbalances suggestive of Gitelman syndrome. Born via C-section at full term, her delivery was complicated by her mother's hypokalemia-induced cardiac arrest. At 8-10 months, she developed a urinary tract infection, with nephrolithiasis diagnosed at 15 months, necessitating a partial nephrectomy for stone removal. Throughout childhood, she frequently experienced urinary tract infections, responding well to sulfamethoxazole and trimethoprim therapy. In adulthood, recurrent severe pyelonephritis episodes ensued. Paresthesias developed, accompanied by intermittent hypokalemia, hypomagnesemia, and occasionally hypocalcemia. Treatment with magnesium and potassium

replacement therapy was initiated. At 40, pancreatitis episodes exacerbated by pyelonephritis and sepsis led to a diabetes diagnosis, prompting a switch from metformin to insulin. A kidney ultrasound at 42 revealed medullary nephrocalcinosis with bilateral renal calculi, non-obstructive. Genetic testing at 43, conducted in November 2019 via an exome-based gene panel, yielded negative results. However, a reanalysis of the ES data in January 2022 identified a VUS in *RRAGD* (Ras-related GTP binding D, HGNC:19903), (NM_021244.4:c.272C>T - p.(Thr91Ile).[1]

Family 2

The index patient was referred at the age of 4 years by her local hospital for management of extensive bilateral stone burden, after presentation with acute abdominal pain. Nephrocalcinosis and stones had been initially identified a year earlier in the context of investigations for recurrent urinary tract infections that started around the age of 2 years. Antenatal history was normal. Parents were consanguineous (first cousins). The stones were initially managed surgically and consisted of calcium phosphate, mainly as carbapatite. Subsequent metabolic analysis showed hypokalemia (3.2 mmol/l), hypomagnesemia (0.57 mmol/l), as well as hypercalciuria (calcium/creatinine ratio 1.66 mol/mol), and initially a clinical diagnosis of Familial Hypomagnesaemia with Hypercalciuria and Nephrocalcinosis was made but could not be confirmed genetically. Family screening showed that her older sister, at the age of 8 years, also had nephrocalcinosis as well as hypokalemia (3.1 mmol/L) and hypomagnesemia (0.59 mmol/L), but no hypercalciuria. Subsequent screening of a new brother at the age of 8 months demonstrated mild nephrocalcinosis but no electrolyte abnormalities. The parents both had

normal electrolytes, as well as normal kidney ultrasounds. An autosomal dominant missense variant was identified in *RRAGD* (NM_021244.4, c.272C>T, p.(Thr91Ile)) by exome sequencing.

Family 3

The female patient presented at the age of 16 years for persistent muscle weakness and cramps upon activities ever since or during infections like tonsillitis. Her mother reported for herself a long history of kidney stones without ever receiving a definite diagnosis. Hypokalemia, hypomagnesemia, and hypocalcemia, together with metabolic alkalosis, were found, and substitution (was prescribed but never continuously taken). An echocardiogram at that time, was normal. An autosomal dominant missense variant was identified in *RRAGD* (NM_021244.4, c.299T>G, p.(Ile100Arg)) by whole exome sequencing. The same mutation was found in the mother. The grandfather was diagnosed in his 30ies initially with Medullary Cystic Kidney disease and currently suffers from CKD IV. He received later in life an implantable defibrillator.

Family 4

A 47-year-old woman was diagnosed with dilated cardiomyopathy (DCM) at age 32, with a baseline left ventricular ejection fraction (LVEF) of 24%, and excessive trabeculation. She had a history of salt-losing tubulopathy and hypomagnesemia since childhood, leading to chronic electrolyte imbalances and nephrocalcinosis, and resembling Bartter syndrome but with negative genetic testing,. Despite treatment, her condition deteriorated, necessitating a cardiac resynchronization therapy-defibrillator (CRT-D) at age 39 due to further LVEF decline and atrial fibrillation (AF). She eventually underwent heart transplantation (HT) after experiencing

advanced heart failure and polymorphic ventricular tachycardia. She developed chronic kidney diseasse (CKD) due to nephrocalcinosis and possibly also to heart failure and its management. The progression of CKD worsened after the heart transplantation, requiring a renal transplantation at age of 42 years. Her monozygotic twin developed in parallel an overlapping phenotype of tubulopathy since pediatric age and DCM leading to HT. Repeated genetic testing for cardiomyopathy and related renal tubulopathies was negative. Several years later, the proband's 6-year-old niece started to develop an incipient phenotype of tubulopathy and excessive ventricular trabeculation that led to a trio exome sequencing that identified the p.(Ser76Leu) variant in RRAGD that was considered as likely pathogenic. Family 4 was first reported by de Frutos et al. (2024).[2]

Family 5

A male patient of 6 months of age was sent to a pediatric center in Buenos Aires, Argentina, with a history of food rejection, intermittent vomiting, hypotonia, and growth retardation. Various clinical investigations were conducted, revealing dilated cardiomyopathy with generalized wall hypomotility and moderate deterioration of left ventricular systolic function. He was evaluated to rule out metabolic and mitochondrial diseases, with all tests returning normal results. At 12 months of age, nephrocalcinosis was detected on a renal ultrasound, initially attributed to the use of loop diuretics. At 11 years of age, the patient was referred to our center for further consultation, and a nephrology evaluation was requested to address the nephrocalcinosis. He had normal kidney function and severe hypomagnesemia and hypokalemia. Renal US showed

medullary nephrocalcinosis, but serum calcium and phosphorus were within normal range, with normal calciuria.

Family 6

The patient is diagnosed of severe hypomagnesemia at 5 years of age. He is asymptomatic but has a history of delayed language development and a slight stature-weight growth delay. He refers chronic polyuria. In the initial study, he presents with normal kidney function, hypokalemia and hypomagnesemia of renal origin, and mild hypercalciuria with normal PTH and vitamin D3 levels. The renal US shows normal-sized kidneys with medullary nephrocalcinosis. The patients showed no pathologic findings in echocardiographic evaluation. He has an older brother who is asymptomatic. The parents have no history of kidney disease.

Family 7

Prematurely born girl, gestational week 32+5, birthweight 1880 g. The oldest of three children to non-consanguineous parents with an unremarkable family history. During infancy, signs of failure to thrive and signs of tubulopathy were apparent, with severe hypomagnesemia and hypokalemia due to renal losses. There were no signs of renal failure or hypertension. The patient was referred to the tertiary care hospital for further diagnostic work-up at the age of 2,5 years. A heart examination had revealed a mitral prolapse and regular echocardiograms had shown no signs of impaired ventricular function. A kidney ultrasound showed medullary nephrocalcinosis. She received supplementation with magnesium and potassium without achieving normalizing of electrolytes. During a summer holiday at the age of seven, she had a varicella infection and was

admitted to hospital due to abdominal pain and fatigue. Investigations revealed dilated cardiomyopathy and severe decompensated heart failure with compromised end organ function, non-responsive to pharmacological heart failure therapy. At the age of 7 years and 8 months, she was listed for heart transplantation and successfully transplanted 4 weeks later. Five years post-transplantation, the patient is in good clinical condition, with excellent graft function and no rejection episodes. Supplementation of magnesium is ongoing but at lower doses. Immunosuppression consists of everolimus and low-dose tacrolimus.

Family 8

The proband is a female, the youngest of five children born to non-consanguineous Ashkenazi Jewish parents. Family history was unremarkable. She was born at 38 weeks of gestation following a pregnancy complicated by severe polyhydramnios (amniotic fluid index [AFI] = 40 cm at 33 weeks of gestation). Her birthweight was 2.835 kg. Following delivery, mild stridor was noted but resolved spontaneously. Development was reportedly normal aside from mild speech delay. At the age of 3 years and 10 months, the proband was admitted to the pediatric intensive care unit (PICU) for evaluation of severe abdominal pain lasting five days. Echocardiography revealed severe dilated cardiomyopathy (DCM) with an ejection fraction (EF) of 16% and severe mitral regurgitation. Laboratory evaluation revealed persistent hypokalemia and hypomagnesemia with significant urinary losses, consistent with a distal tubulopathy. Renal ultrasound showed marked nephrocalcinosis. Infectious workup was negative. The combination of severe prenatal polyhydramnios, cardiomyopathy, and renal tubular dysfunction raised clinical suspicion for an

underlying genetic disorder. Trio whole-genome sequencing was performed, revealing a heterozygous de novo variant in RRAGD: p.(Ser76Leu), which has previously been reported as pathogenic (Schlingmann et al., 2021). The patient is currently being treated with enalapril, carvedilol, spironolactone, hydrochlorothiazide, and digoxin, along with potassium and magnesium supplementation. Under this regimen, her ejection fraction has improved to 36%.

Family 9

Family 9 was previously described in Schlingmann *et al.,* (2021).[1]

Supplementary table S1. List of mutagenesis primers.

Usage	Variants	Primer name	Sequence (5'-3')
Subcloning	All variants	Forward	GCCGGTACCAGCCAGGTGCTGG
		Reverse	GCCCTCGAGCTACAGCAGCACTCTAGG
Mutagenesis	h*RRAGD* p.(Ser77Phe)	Forward	GGAGAAGCGGCAAGTCGTTTATTCAGAAAGTTGTC
		Reverse	GACAACTTTCTGAATAAACGACTTGCCGCTTCTCC
	h*RRAGD* p.(Thr91Ile)	Forward	CACAAAATGTCTCCCAACGAAATTCTGTTCTTGGAGAGCAC
		Reverse	GTGCTCTCCAAGAACAGAATTTCGTTGGGAGACATTTTGTG
	h*RRAGD* p.(Ile100Arg)	Forward	GAGAGCACTAATAAGAGATGCCGGGAAGATGTTTC
		Reverse	GAAACATCTTCCCGGCATCTCTTATTAGTGCTCTC

Supplementary table S2. Furosemide treatment. Values represent the median [min; max] of four ADKH-*RRAGD* patients following 3-hr p.o. 40 mg furosemide. T=0: baseline, T=1, 2, 3: number of hours post-furosemide treatment.

	T=0	T=1	T=2	T=3
Blood				
Osmolarity (mosm/kg; N=281-303)	285.5 [284; 287]	284 [282; 287]	281.5 [277; 285]	277.5 [273; 281]
Na^+ (mmol/L; N=136-145)	141 [140; 142]	141.5 [141; 142]	139.5 [138; 141]	139 [137; 140]
K^+ (mmol/L; N=3.5-5.1)	3.42 [2.96; 3.55]	3.42 [3.23; 3.6]	3.4 [3.13; 3.64]	3.22 [2.97; 3.4]
Cl^- (mmol/L; N=98-107)	97 [94; 101]	95 [90; 98]	91 [88; 95]	91 [86; 94]
Ca^{2+} (mmol/L; N=2.2-2.6)	2.3 [2.26; 2.44]	2.44 [2.32; 2.53]	2.45 [2.37; 2.62]	2.36 [2.29; 2.53]
Mg^{2+} (mmol/L; N=0.66-1.07)	0.44 [0.4; 0.47]	0.44 [0.4; 0.47]	0.38 [0.37; 0.41]	0.37 [0.36; 0.41]
HCO3 (mmol/L; N=22-31)	33.4 [31.7; 35]	35.1 [31.5; 37.1]	34.35 [31.8; 38.1]	35.1 [30.2; 36.7]
Creatinine (mg/dL; N=0.73-1.18)	0.765 [0.51; 0.93]	0.74 [0.43; 0.86]	0.75 [0.46; 0.91]	0.75 [0.42; 0.88]
Urine				
Osmolarity (mosm/kg; N=50-1200)	439.5 [140; 507]	221.5 [161; 271]	214 [177; 258]	176 [135; 226]
Na^+ (mmol/L; N=22.3-200.1)	52.5 [33; 90]	81.5 [60; 105]	90 [76; 103]	58 [50; 82]
K^+ (mmol/L; N=20.6-101.9)	52.15 [11.8; 60.3]	12.95 [9.4; 17.5]	10.75 [7.9; 17.7]	15.95 [9.3; 19.1]
Cl^- (mmol/L; N=27-225)	59.5 [33; 132]	95.5 [67; 113]	101.5 [84; 120]	76.5 [60; 99]
Creatinine (mg/dL; N=0-37.7)	0.7 [0.13; 1.89]	0.1 [0.05; 0.18]	0.06 [0.03; 0.08]	0.1 [0.03; 0.16]
Ca/Creat (mmol/g creat; N=0.3-6.1)	2.64 [0.46; 2.92]	10.1 [6.38; 12.88]	18.98 [14.17; 27.67]	9.9 [6.6; 19.71]
Mg/Creat (mmol/g creat; N=0.74-4.53)	2.805 [1.71; 5.85]	6.15 [5.68; 12.88]	12.7 [9.4; 21.67]	7.47 [5.33; 17.35]
Fractional excretions				
Na^+ (%; N=0.1-2)	0.705 [0.1; 0.99]	4.3 [2.37; 5.05]	8.43 [7.17; 10.76]	4.03 [1.99; 4.66]
K^+ (%; N=5.5-17)	14.55 [6.6; 26]	25.15 [17.6; 37.8]	45.6 [34.1; 58.9]	39.9 [23.4; 46.5]
Cl^- (%; N=0.7-2)	1.0 [0.2; 2.05]	7.0 [4.32; 8.54]	14.3 [13.39; 18.41]	7.3 [4.28; 9.03]
Ca^{2+} (%; N=1-2.6)	0.78 [0.14; 1.1]	2.84 [1.78; 3.5]	5.3 [4.89; 7.09]	3.18 [1.91; 3.86]
Mg^{2+} (%; N=3-5)	5.6 [2.5; 6.5]	11.5 [8.3; 11.6]	24.35 [19.6; 25.3]	15.3 [9.1; 19.2]

Supplementary table S3. HCT treatment. Values represent the median [min; max] of four ADKH-*RRAGD* patients following 6-hr p.o. 50 mg HCT. T=0: baseline, T=2, 4, 6: number of hours post-HCT treatment

	T=0	T=2	T=4	T=6
Blood				
Osmolarity (mosm/kg; N=281-303)	287.5 [281; 291]	282 [277; 283]	281 [278; 285]	279.5 [275; 282]
Na^+ (mmol/L; N=136-145)	141.5 [140; 144]	140 [139; 141]	139 [138; 141]	139.5 [138; 140]
K^+ (mmol/L; N=3.5-5.1)	3.26 [3.22; 3.7]	3.63 [3.2; 4.03]	3.24 [2.99; 3.67]	3.34 [3.05; 3.65]
Cl^- (mmol/L; N=98-107)	96 [96; 103]	93.5 [92; 98]	93 [92; 98]	92.5 [90; 96]
Ca^{2+} (mmol/L; N=2.2-2.6)	2.38 [2.26; 2.63]	2.46[2.36; 2.65]	2.47 [2.39; 2.71]	2.54 [2.44; 2.79]
Mg^{2+} (mmol/L; N=0.66-1.07)	0.43 [0.39; 0.48]	0.47 [0.41; 0.49]	0.44 [0.37; 0.49]	0.44 [0.39; 0.52]
HCO3 (mmol/L; N=22-31)	32.4 [30.9; 34.8]	33.05 [30.7; 35.1]	34.45 [32.9; 37.4]	35.55 [32.3; 37.8]
Creatinine (mg/dL; N=0.73-1.18)	0.75 [0.58; 1.04]	0.73 [0.54; 0.98]	0.73 [0.55; 1]	0.75 [0.54; 0.94]
Urine				
Osmolarity (mosm/kg; N=50-1200)	351 [112; 640]	219 [117; 323]	140 [113; 211]	113 [95; 215]
Na^+ (mmol/L; N=22.3-200.1)	50.5 [14; 115]	66 [36; 100]	44 [25; 70]	34.5 [24; 66]
K^+ (mmol/L; N=20.6-101.9)	40.5 [11.9; 53.6]	18.05 [11.1; 25.8]	13.4 [6.5; 14.9]	11 [7.3; 15.2]
Cl^- (mmol/L; N=27-225)	71 [23; 129]	71.5 [36; 128]	41 [32; 86]	34.5 [30; 82]
Creatinine (mg/dL; N=0-37.7)	9.25 [6.8; 11.9]	6.35 [5.9; 8.2]	7.5 [3.9; 9.6]	7.35 [5; 9.4]
Ca/Creat (mmol/g creat; N=0.3-6.1)	1.13 [0.35; 1.59]	0.4 [0.23; 0.55]	0.17 [0.12; 0.21]	0.16 [0.11; 0.25]
Mg/Creat (mmol/g creat; N=0.74-4.53)	1.86 [0.45; 4.74]	0.79 [0.35; 1.39]	0.5 [0.34; 0.69]	0.45 [0.42; 0.74]
Fractional excretions				
Na^+ (%; N=0.1-2)	0.49 [0.17; 1.26]	2.13 [2.01; 2.54]	2.085 [1.11; 3.28]	1.84 [1.47; 2.71]
K^+ (%; N=5.5-17)	17.15 [6.3; 25.5]	22.65 [19.2; 34]	22.45 [17.4; 29.8]	21.95 [19.8; 31.1]
Cl^- (%; N=0.7-2)	1.19 [0.31; 2.1]	3.7 [3.15; 4.12]	3.45 [2.04; 4.65]	3.18 [2.68; 4.02]
Ca^{2+} (%; N=1-2.6)	0.64 [0.29; 0.96]	0.77 [0.61; 0.96]	0.48 [0.24; 0.59]	0.54 [0.18; 0.82]
Mg^{2+} (%; N=3-5)	5.05 [2.9; 9.9]	7.9 [6.6; 9]	8.15 [4.8; 8.9]	8.25 [7; 9]

```
S. cerevisiae      ------------------------------------------------------------   0
C. elegans         ------------------------------------------------------------   0
D. melanogaster    ----------------------------MSYDDDDYPAD----------------TFP--   14
D. rerio           MTS----AR-----KNNEVDGENEALSFDYYDDDDDFDAFTDGDG---------------   36
X. tropicalis      -MSLPGKSH-ERQQEEEGDDEDDDIMGVSDYGDGDSFMDGERGS----------------   42
M. musculus        MSQVLGKPQPQGEDGGE-DQEEDELVGLAGYEDGPESSDAELDSGPEEGESRRNSWMPRS   59
H. sapiens         MSQVLGKPQPQDEDDAEEEEEEDELVGLADYGDGPDSSDADPDSGTEEG-----------   49

S. cerevisiae      -----------------------------------------------MSLEATDSKAMVL   13
C. elegans         -----------------------------MESDPDEDFDDYRYGIDE-EDDYPDSRPTVI   30
D. melanogaster    -----------------------KDFGY---RA--YNQDGL--ELEPNATGSSETKPRIL   44
D. rerio           --------------------------------D--CSCDGG--VLGFSDPFSSEVKPRIL   60
X. tropicalis      ------------------------------------EGDDE--VLDFTDPFSTEVKPRIL   64
M. musculus        WCSEATRHECWEPGLWRSSHLLGIGGGWRMLRR--QRQADF--FLDFSDPFSTEVKPRIL   115
H. sapiens         -------------------------------------------VLDFSDPFSTEVKPRIL   66
                                                                :  :  ::

                       G1                      G2                     G3
S. cerevisiae      LMGVRRCGKSSICKVVFHNMQPLDTLYLESTSNPSLEHF--STLIDLAVMELPGQLNYFE   71
C. elegans         LMGHKRSGKTSIRKVVFQKMSPNETMFVESTARITRDTI-CSSFINFETIEFPGQMCPFD   89
D. melanogaster    LMGMRRSGKSSIQKVVFHKMSPNETLFLESTSKIVKDDINNSSFVQFQIWDFPGQIDFFE   104
D. rerio           LMGLRRSGKSSIQKVVFHKMSPNETLFLESTNKICREDVSNSSFVSFQIWDFPGQIDFFD   120
X. tropicalis      LMGLRRSGKSSIQKVVFHKMSPNETLFLESTNKICREDVSNSSFVNFQIWDFPGQIDFFD   124
M. musculus        LMGLRRSGKSSIQKVVFHKMSPSETLFLESTNRICREDVSNSSFVNFQIWDFPGQIDFFD   175
H. sapiens         LMGLRRSGKSSIQKVVFHKMSPNETLFLESTNKICREDVSNSSFVNFQIWDFPGQIDFFD   126
                   *** :*.**:** ****::*.* :*:::*** .    : .  *:::.:   ::***:  *:

                                                                   G4
S. cerevisiae      PSYDSERLFKSVGALVYVIDSQDEYINAITNLAMIIEYAYKVNPSINIEVLIHKVDGLSE   131
C. elegans         DSLDPVGVFQKCEALLFIIDAQAELQEPIATLVEYFCRAYKINQNIKFEVFVHKADGLTE   149
D. melanogaster    PTFDSDMIFGGCGALVFVIDAKDDYNEALTKFKNTVLQAYKVNKRIKFEVFIHKVDGISD   164
D. rerio           PTFDYEMIFRGTGALIFVIDSQDDYVEALSRLHLTVTRAYKVNPDINFEVFIHKVDGLSD   180
X. tropicalis      PTFDYEMIFRGTGALIFVIDSQDDYMEALARLHLTVTSAYKVNPDINFEVFIHKVDGLSD   184
M.musculus         PTFDYEMIFRGTGALIFVIDSQDDYMEALARLHLTVTRAYKVNTDINFEVFIHKVDGLSD   235
H. sapiens         PTFDYEMIFRGTGALIFVIDSQDDYMEALARLHLTVTRAYKVNTDINFEVFIHKVDGLSD   186
                    : *   :*    **:::**:: :  : :: :    .  ***:*  *::**::**.**:::

                                                G5
S. cerevisiae      DFKVDAQRDIMQRTGEELLELGLDGVQVSFYLTSIFDHSIYEAFSRIVQKLIPELSFLEN   191
C. elegans         EARVETKFNIYHQVKETIKDQIDVDLQVTYHLTSIYDHSIFEAFSKVVQNLVKQLPTLER   209
D. melanogaster    DSKMESQRDIHQRSSDDLNEAGLDQIHLSFHLTSIYDHSIFEAFSKVVQKLIPQLPTLEN   224
D. rerio           DHKIEKQRDIHKRANDDLADAGLERIHLSFYLTSIYDHSIFEAFSKVVQKLIPQLPTLEN   240
X. tropicalis      DHKIETQRDIHQRANDDLVDAGLEKIHLSFYLTSIYDHSIFEAFSKVVQKLIPQLPTLEN   244
M. musculus        DHKIETQRDIHQRANDDLADAGLEKIHLSFYLTSIYDHSIFEAFSKVVQKLIPQLPTLEN   295
H. sapiens         DHKIETQRDIHQRANDDLADAGLEKIHLSFYLTSIYDHSIFEAFSKVVQKLIPQLPTLEN   246
                   : ::: : :* ::  : : :     ::::::****:****:****::**:*: :*  **.

S. cerevisiae      MLDNLIQHSKIEKAFLFDVNSKIYVSTDSNPVDIQMYEVCSEFIDVTIDLFDLYKAPVLR   251
C. elegans         LLDVFNNSSKVTKSFLFDILSKIYIATDSEPVEMSIYELCCDMIDVTLDLSSIYGVAEN-   268
D. melanogaster    LLNIFIPNSGIEKAFLFDVVSKIYIATDSSPVDMQTYELCCDMIDVVIDLSSIYSSE---   281
D. rerio           LLNIFISNSGIEKAFLFDVVSKIYIATDSSPVDMQTYELCCDMIDVVIDISCIYGLSGDE   300
X. tropicalis      LLNIFISNSGIEKVFLFDVVSKIYIATDSSPVDMQTYELCCDMIDVVIDISCIYGLE--G   302
M. musculus        LLNIFISNSGIEKAFLFDVVSKIYIATDSTPVDMQTYELCCDMIDVVIDISCIYGLKEDG   355
H. sapiens         LLNIFISNSGIEKAFLFDVVSKIYIATDSTPVDMQTYELCCDMIDVVIDISCIYGLKEDG   306
                   :*: :   * : * ****: ****::*** **::. **:*.::***.:*:  :*

S. cerevisiae      NSQKSSDKDNVINPRNELQNVSQLANGVIIYLRQMIRGLALVAIIRPNGTDMESCLTVAD   311
C. elegans         -GS---------NYDERSSSVIRLKSEQVMFLRQVNKHLALVFIMKEDGNEKA---GFID   315
D. melanogaster    -ET---------AFDSGSSSLIKLNNNTILYLREVNKFLALVCILREENFNRQ---GVID   328
D. rerio           GGT---------PYDKESMAIIHLNNTTVMYLKEVTKFLALVCFLREESFEKK---GLID   348
X. tropicalis      AGT---------PYDKESLAIIKLNNTTVLYLKEVTKFLALVCFVREESFERK---GLID   350
M. musculus        AGA---------PYDKDSTAIIKLNNTTVLYLKEVTKFLALVCFVREESFERK---GLID   403
H. sapiens         AGT---------PYDKESTAIIKLNNTTVLYLKEVTKFLALVCFVREESFERK---GLID   354
                                  .    : :* .  :::*::: : **** ::: :. :      . *

S. cerevisiae      YNIDIFKKGLEDIWANARASQAKNSIEDDV----------------------------   341
C. elegans         HNFGVFKAGIEQVFKVKNRGVNF-----------------------------------   338
D. melanogaster    YNFICFRDAISEVFELRLKRQKQLENNDQDDDDLVDEQTLIRHGHDAGGISRAQPIN   385
D. rerio           YNFHCFKKAIEEVFDVRLKVQRSRKLLSQRRWS---------RQTVPNGTQVLP-H-   394
X. tropicalis      YNFHCFRKAIQEVFEVRVKVLRSRKHQSQTKKS---------RRATPNGTPGVP-L-   396
M. musculus        YNFHCFRKAIHEVFEVRMKMVKSRKAQSRLPKK---------TGATPNGTPRVL-L-   449
H. sapiens         YNFHCFRKAIHEVFEVRMKVVKSRKVQNRLQKK---------KRATPNGTPRVL-L-   400
                   :*:  *: .: :::
```

```
S. cerevisiae     ------------------------------------------------------------    0
C. elegans        ------------------------------------------------------------    0
D. melanogaster   ----------------------------MSYDDDDYPAD----------------TFP--    14
D. rerio          MTS----AR-----KNNEVDGENEALSFDYYDDDDDFDAFTDGDG---------------    36
X. tropicalis     -MSLPGKSH-ERQQEEEGDDEDDDIMGVSDYGDGDSFMDGERGS----------------    42
M. musculus       MSQVLGKPQPQGEDGGE-DQEEDELVGLAGYEDGPESSDAELDSGPEEGESRRNSWMPRS    59
H. sapiens        MSQVLGKPQPQDEDDAEEEEEEDELVGLADYGDGPDSSDADPDSGTEEG-----------    49

S. cerevisiae     -----------------------------------------------MSLEATDSKAMVL    13
C. elegans        -----------------------------MESDPDEDFDDYRYGIDE-EDDYPDSRPTVI    30
D. melanogaster   -----------------------KDFGY---RA--YNQDGL--ELEPNATGSSETKPRIL    44
D. rerio          --------------------------------D--CSCDGG--VLGFSDPFSSEVKPRIL    60
X. tropicalis     ------------------------------------EGDDE--VLDFTDPFSTEVKPRIL    64
M. musculus       WCSEATRHECWEPGLWRSSHLLGIGGGWRMLRR--QRQADF--FLDFSDPFSTEVKPRIL    115
H. sapiens        -------------------------------------------VLDFSDPFSTEVKPRIL    66
                                                                   : :  ::

                      G1                   G2                    G3
S. cerevisiae     LMGVRRCGKSSICKVVFHNMQPLDTLYLESTSNPSLEHF--STLIDLAVMELPGQLNYFE    71
C. elegans        LMGHKRSGKTSIRKVVFQKMSPNETMFVESTARITRDTI-CSSFINFETIEFPGQMCPFD    89
D. melanogaster   LMGMRRSGKSSIQKVVFHKMSPNETLFLESTSKIVKDDINNSSFVQFQIWDFPGQIDFFE    104
D. rerio          LMGLRRSGKSSIQKVVFHKMSPNETLFLESTNKICREDVSNSSFVSFQIWDFPGQIDFFD    120
X. tropicalis     LMGLRRSGKSSIQKVVFHKMSPNETLFLESTNKICREDVSNSSFVNFQIWDFPGQIDFFD    124
M. musculus       LMGLRRSGKSSIQKVVFHKMSPSETLFLESTNRICREDVSNSSFVNFQIWDFPGQIDFFD    175
H. sapiens        LMGLRRSGKSSIQKVVFHKMSPNETLFLESTNKICREDVSNSSFVNFQIWDFPGQIDFFD    126
                  *** :*.**:** ****::*.* :*:::*** .    : .  *:::.:   ::***:  *:

                                                                    G4
S. cerevisiae     PSYDSERLFKSVGALVYVIDSQDEYINAITNLAMIIEYAYKVNPSINIEVLIHKVDGLSE    131
C. elegans        DSLDPVGVFQKCEALLFIIDAQAELQEPIATLVEYFCRAYKINQNIKFEVFVHKADGLTE    149
D. melanogaster   PTFDSDMIFGGCGALVFVIDAKDDYNEALTKFKNTVLQAYKVNKRIKFEVFIHKVDGISD    164
D. rerio          PTFDYEMIFRGTGALIFVIDSQDDYVEALSRLHLTVTRAYKVNPDINFEVFIHKVDGLSD    180
X. tropicalis     PTFDYEMIFRGTGALIFVIDSQDDYMEALARLHLTVTSAYKVNPDINFEVFIHKVDGLSD    184
M.musculus        PTFDYEMIFRGTGALIFVIDSQDDYMEALARLHLTVTRAYKVNTDINFEVFIHKVDGLSD    235
H. sapiens        PTFDYEMIFRGTGALIFVIDSQDDYMEALARLHLTVTRAYKVNTDINFEVFIHKVDGLSD    186
                   : *   :*    **:::**:: :  : :: :   .  ***:*  *::**::**.**:::

                                              G5
S. cerevisiae     DFKVDAQRDIMQRTGEELLELGLDGVQVSFYLTSIFDHSIYEAFSRIVQKLIPELSFLEN    191
C. elegans        EARVETKFNIYHQVKETIKDQIDVDLQVTYHLTSIYDHSIFEAFSKVVQNLVKQLPTLER    209
D. melanogaster   DSKMESQRDIHQRSSDDLNEAGLDQIHLSFHLTSIYDHSIFEAFSKVVQKLIPQLPTLEN    224
D. rerio          DHKIEKQRDIHKRANDDLADAGLERIHLSFYLTSIYDHSIFEAFSKVVQKLIPQLPTLEN    240
X. tropicalis     DHKIETQRDIHQRANDDLVDAGLEKIHLSFYLTSIYDHSIFEAFSKVVQKLIPQLPTLEN    244
M. musculus       DHKIETQRDIHQRANDDLADAGLEKIHLSFYLTSIYDHSIFEAFSKVVQKLIPQLPTLEN    295
H. sapiens        DHKIETQRDIHQRANDDLADAGLEKIHLSFYLTSIYDHSIFEAFSKVVQKLIPQLPTLEN    246
                  : ::: : :* ::  : : :     ::::::****:****:****::**:*: :*  **.

S. cerevisiae     MLDNLIQHSKIEKAFLFDVNSKIYVSTDSNPVDIQMYEVCSEFIDVTIDLFDLYKAPVLR    251
C. elegans        LLDVFNNSSKVTKSFLFDILSKIYIATDSEPVEMSIYELCCDMIDVTLDLSSIYGVAEN-    268
D. melanogaster   LLNIFIPNSGIEKAFLFDVVSKIYIATDSSPVDMQTYELCCDMIDVVIDLSSIYSSE---    281
D. rerio          LLNIFISNSGIEKAFLFDVVSKIYIATDSSPVDMQTYELCCDMIDVVIDISCIYGLSGDE    300
X. tropicalis     LLNIFISNSGIEKVFLFDVVSKIYIATDSSPVDMQTYELCCDMIDVVIDISCIYGLE--G    302
M. musculus       LLNIFISNSGIEKAFLFDVVSKIYIATDSTPVDMQTYELCCDMIDVVIDISCIYGLKEDG    355
H. sapiens        LLNIFISNSGIEKAFLFDVVSKIYIATDSTPVDMQTYELCCDMIDVVIDISCIYGLKEDG    306
                  :*: :   * : * ****: ****::*** **::. **:*.::***.:*:  :*

S. cerevisiae     NSQKSSDKDNVINPRNELQNVSQLANGVIIYLRQMIRGLALVAIIRPNGTDMESCLTVAD    311
C. elegans        -GS---------NYDERSSSVIRLKSEQVMFLRQVNKHLALVFIMKEDGNEKA---GFID    315
D. melanogaster   -ET---------AFDSGSSSLIKLNNNTILYLREVNKFLALVCILREENFNRQ---GVID    328
D. rerio          GGT---------PYDKESMAIIHLNNTTVMYLKEVTKFLALVCFLREESFEKK---GLID    348
X. tropicalis     AGT---------PYDKESLAIIKLNNTTVLYLKEVTKFLALVCFVREESFERK---GLID    350
M. musculus       AGA---------PYDKDSTAIIKLNNTTVLYLKEVTKFLALVCFVREESFERK---GLID    403
H. sapiens        AGT---------PYDKESTAIIKLNNTTVLYLKEVTKFLALVCFVREESFERK---GLID    354
                                 .    : :* .  :::*::: : **** ::: :. :      . *

S. cerevisiae     YNIDIFKKGLEDIWANARASQAKNSIEDDV----------------------------    341
C. elegans        HNFGVFKAGIEQVFKVKNRGVNF-----------------------------------    338
D. melanogaster   YNFICFRDAISEVFELRLKRQKQLENNDQDDDDLVDEQTLIRHGHDAGGISRAQPIN    385
D. rerio          YNFHCFKKAIEEVFDVRLKVQRSRKLLSQRRWS---------RQTVPNGTQVLP-H-    394
X. tropicalis     YNFHCFRKAIQEVFEVRVKVLRSRKHQSQTKKS---------RRATPNGTPGVP-L-    396
M. musculus       YNFHCFRKAIHEVFEVRMKMVKSRKAQSRLPKK---------TGATPNGTPRVL-L-    449
H. sapiens        YNFHCFRKAIHEVFEVRMKVVKSRKVQNRLQKK---------KRATPNGTPRVL-L-    400
                  :*:  *: .: :::
```

Supplementary figure S1. Alignment of RagD sequences showed conservation of variant sites. Multiple alignment analysis of RagD protein sequences in yeast (Saccharomyces cerevisiae; P53290), round worm (Caenorhabditis elegans; G5EGB3), fruit fly (Drosophila melanogaster; Q7K519), zebrafish (Danio rerio; F1QIB5), western clawed frog (Xenopus tropicalis; Q0VFL0), mouse (Mus musculus; Q7TT45), and human (Homo sapiens; Q9NQL2). GTP-binding domains, or G-boxes, are highlighted in blue. Affected residues by variants described in this study are highlighted in purple. Residues that have been previously described are highlighted in orange.

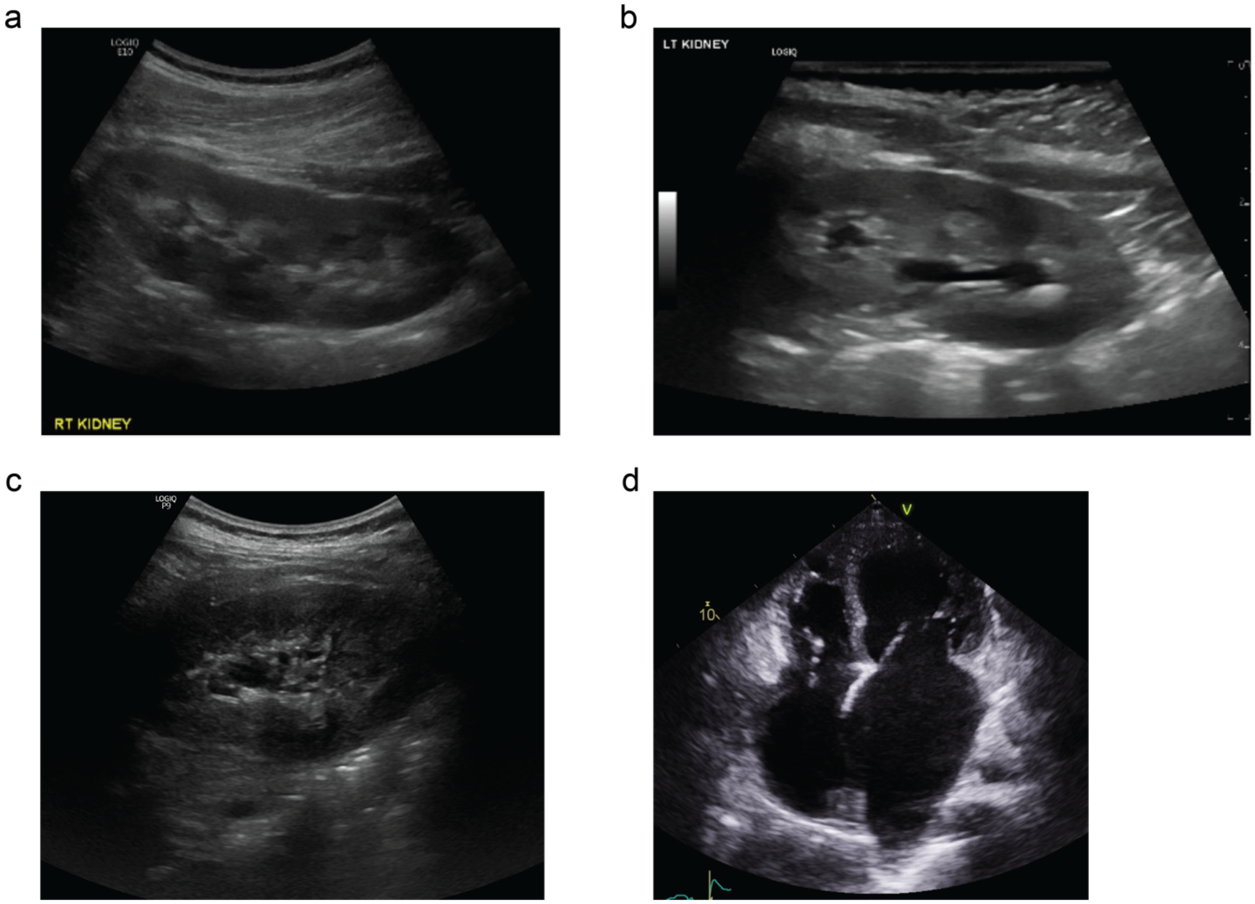

Supplementary figure S2. Ultrasound images of patients with pathogenic *RRAGD* variants. (a-c) Ultrasound images of the kidney of patients (a) F2.II.1, (b) F2.II.3, and (c) F3.III.1. (d) Apical 4 chamber view of heart ultrasound in family F4.

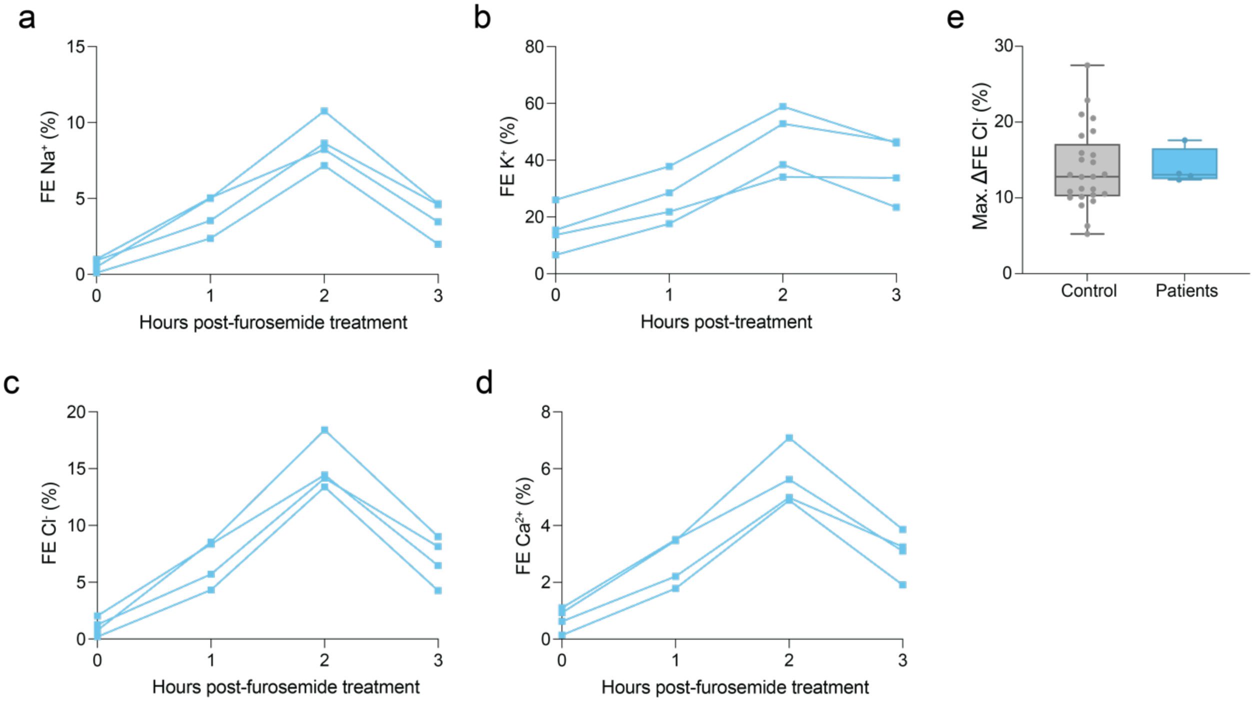

Supplementary figure S3. Fractional excretions in ADKH-*RRAGD* patients following furosemide treatment. Fractional excretions (FE) of (a) Na^+, (b) K^+, (c) Cl^-, and (d) Ca^{2+} at 0, 1, 2, and 3 hr post-p.o. 40 mg furosemide administration. (e) Maximal Δ FE Cl^- (*i.e.,* max. FE Cl^- – baseline value) of healthy individuals (N=25, grey) and ADKH-*RRAGD* patients (N=4, blue). (a-e) Data points indicate individuals. (e) the boxes range from the 25th to the 75th quartile, the whiskers extend from max. to min. points, and the middle lines indicate the median.

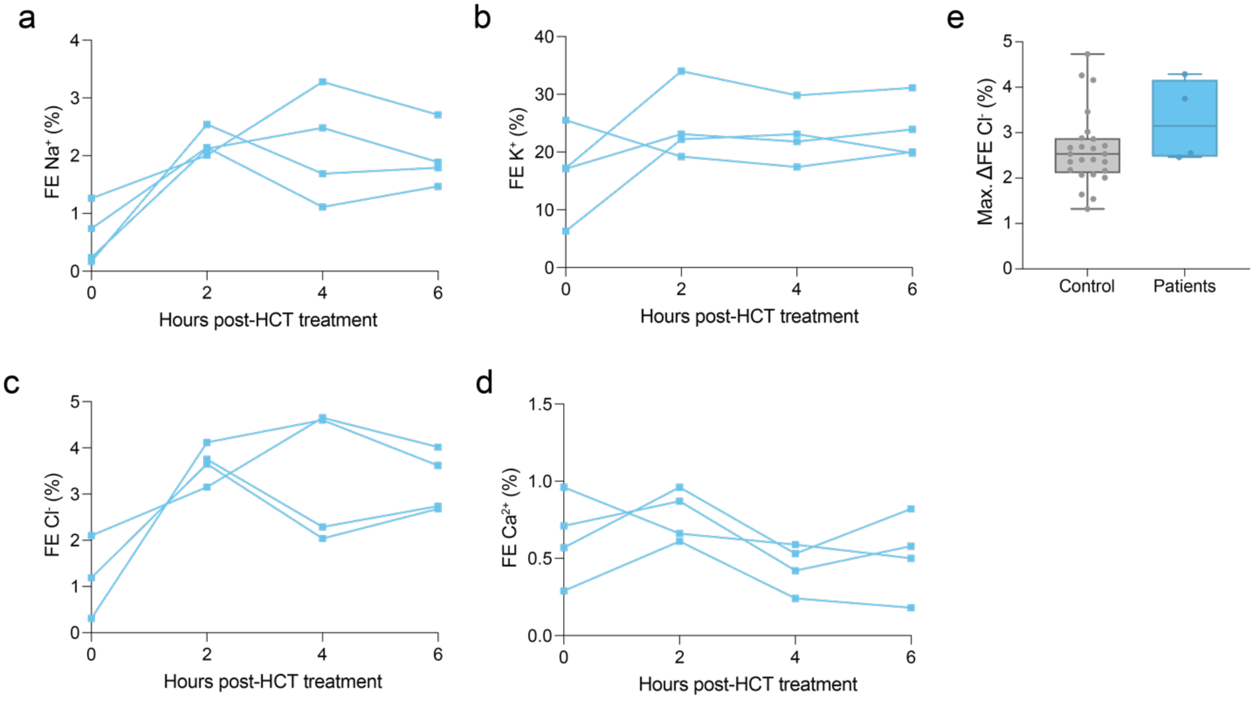

Supplementary figure S4. Fractional excretions in ADKH-*RRAGD* patients following HCT treatment. Fractional excretions (FE) of (a) Na^+, (b) K^+, (c) Cl^-, and (d) Ca^{2+} at 0, 2, 4, and 6 hr post-p.o. 50 mg hydrochlorothiazide (HCT) administration. (e) Maximal Δ FE Cl^- (*i.e.,* max. FE Cl^- – baseline value) of healthy individuals (N=25, grey) and ADKH-*RRAGD* patients (N=4, blue). (a-e) Data points indicate individuals. (e) the boxes range from the 25th to the 75th quartile, the whiskers extend from max. to min. points, and the middle lines indicate the median.

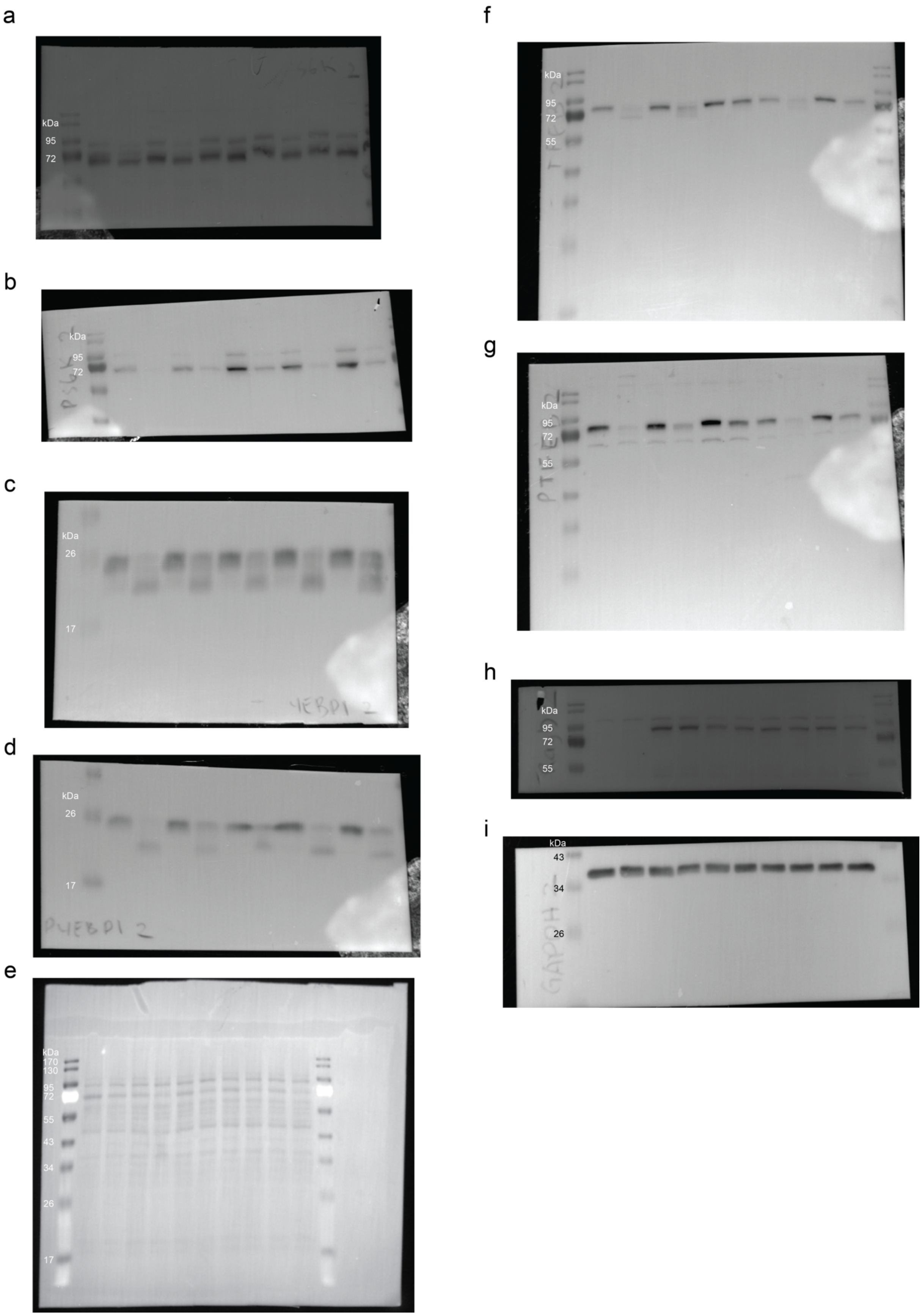
a
kDa
95
72
b
kDa
95
72
c
kDa
26
17
4EBP1 2
d
kDa
26
17
P4EBP1 2
e
kDa
170
130
95
72
55
43
34
26
17
f
kDa
95
72
55
g
kDa
95
72
55
h
kDa
95
72
55
i
kDa
43
34
26

Supplementary figure S5. Full uncropped immunoblots. Unaltered images of immunoblots merged with illuminated marker of (a) S6K, (b) phospho-S6K, (c) 4E-BP1, (d) phospho-4E-BP1, (e) ponceau S scan, (f) TFEB, (g) phospho-TFEB, (h) GFP, and (i) GAPDH.

Supplementary References

S1. Wilke M, Klee EW, Dhamija R, et al. Diagnostic yield of exome and genome sequencing after non-diagnostic multi-gene panels in patients with single-system diseases. *Orphanet J Rare Dis*. May 24 2024;19(1):216. doi:10.1186/s13023-024-03213-x

S2. de Frutos F, Diez-Lopez C, Garcia-Romero E, et al. Dilated Cardiomyopathy With Concomitant Salt-Losing Renal Tubulopathy Caused by Heterozygous RRAGD Gene Variant. *Circ Genom Precis Med*. Apr 2024;17(2):e004336. doi:10.1161/CIRCGEN.123.004336

S3. Pejaver V, Byrne AB, Feng BJ, et al. Calibration of computational tools for missense variant pathogenicity classification and ClinGen recommendations for PP3/BP4 criteria. *Am J Hum Genet*. Dec 1 2022;109(12):2163-2177. doi:10.1016/j.ajhg.2022.10.013

S4. Adzhubei IA, Schmidt S, Peshkin L, et al. A method and server for predicting damaging missense mutations. *Nat Methods*. Apr 2010;7(4):248-9. doi:10.1038/nmeth0410-248
